# Can AI assess literature like experts? An entropy-based comparison of ChatGPT-4o, DeepSeek R1, and human ratings

**DOI:** 10.3389/frma.2025.1684137

**Published:** 2025-11-10

**Authors:** Yichen Zhou, Haixu Hu

**Affiliations:** School of Sport Training, Nanjing Sport Institute, Nanjing, Jiangsu, China

**Keywords:** artificial intelligence (AI), expert assessment, literature evaluation, ChatGPT-4o, DeepSeek R1, entropy-based method, machine and human comparison

## Abstract

**Background:**

Manual quality assessment of systematic reviews is labor-intensive, time-consuming, and subject to reviewer bias. With recent advances in large language models (LLMs), it is important to evaluate their reliability and efficiency as potential replacements for human reviewers.

**Aim:**

This study assessed whether generative AI models can substitute for manual reviewers in literature quality assessment by examining rating consistency, time efficiency, and discriminatory performance across four established appraisal tools.

**Methods:**

Ninety-one systematic reviews were evaluated using AMSTAR 2, CASP, PEDro, and RoB 2 by both human reviewers and two LLMs (ChatGPT-4.0 and DeepSeek R1). Entropy-based indicators quantified rating consistency, while Spearman correlations, receiver operating characteristic (ROC) analysis, and processing-time comparisons were used to assess the relationship between time variability and scoring reliability.

**Results:**

The two LLMs demonstrated high consistency with human ratings (mean entropy = 0.42), with particularly strong alignment for PEDro (0.17) and CASP (0.25). Average processing time per article was markedly shorter for LLMs (33.09 s) compared with human reviewers (1,582.50 s), representing a 47.80-fold increase in efficiency. Spearman correlation analysis showed a statistically significant positive association between processing-time variability and rating entropy (ρ = 0.24, *p* = 0.026), indicating that greater time variability was associated with lower consistency. ROC analysis further showed that processing-time variability moderately predicted moderate-to-low consistency (AUC = 0.65, *p* = 0.045), with 46.00 seconds identified as the optimal cutoff threshold.

**Conclusion:**

LLMs markedly reduce appraisal time while maintaining acceptable rating consistency in literature quality assessment. Although human validation is recommended for cases with high processing-time variability (>46.00 s), generative AI represents a promising approach for standardized, efficient, and scalable quality appraisal in evidence synthesis.

## 1 Introduction

Systematic reviews and meta-analyses, as essential methods for synthesizing existing research evidence, have gradually evolved into an indispensable methodological paradigm in interdisciplinary research. Leading journals such as *Nature* (Momeni et al., 2025) and *Science* (Hagiwara et al., 2024) frequently publish related work, as the reliability of secondary analyses critically depends on the methodological quality of the included studies. Without rigorous quality appraisal, low-quality evidence may be incorporated into reviews, thereby leading to distorted conclusions—“garbage in, garbage out”. At present, most quality assessments rely on researchers' subjective judgement using established checklists such as PRISMA, AMSTAR, or GRADE. This manual process is labor-intensive, prone to cognitive bias, and inefficient when handling large evidence bases or when cross-disciplinary expertise is required. Moreover, quality appraisal is inherently repetitive and resource-demanding, making it a suitable target for automation (Giummarra et al., 2020). With the rapid advancement of LLMs, AI systems such as ChatGPT and DeepSeek have shown considerable potential across multiple stages of evidence synthesis. These stages include formulating clinical questions, conducting literature searches, screening studies, extracting data, and refining textual outputs. By supporting these processes, LLMs can reduce workload, improve efficiency, and optimize resource allocation (Luo et al., 2024a), thereby emerging as promising tools to facilitate and streamline evidence synthesis.

Recent evidence confirms that LLMs can already outperform manual reviewers during study selection. When applied to diagnostic research, LLMs have shown promising performance in literature screening. Using a meta-prompt, GPT-3.5-turbo and GPT-4 achieved very high sensitivity in screening titles and abstracts (0.988 and 0.982, respectively), with GPT-4 further improving specificity to 0.740 (Kataoka et al., 2023). In a subsequent evaluation, GPT-4-Turbo combined a PICO-based prompt with study-design filters to screen 17,000 records addressing five clinical questions, yielding a sensitivity of 0.91 (95% CI 0.77–0.97) and a specificity of 0.98 (95% CI 0.96–0.99), while reducing processing time from 17.2 to 1.3 min per 100 papers (Oami et al., 2024). Another study employed a Python-based GPT API pipeline to triage 24,307 citations, reporting an overall accuracy of 0.91, sensitivities of 0.91 for exclusions and 0.76 for inclusions, a macro-F1 of 0.66, and a PABAK of 0.96, compared with κ = 0.46 for dual human review (Guo et al., 2024). Compared with conventional evidence mapping, AI-assisted approaches substantially improve the efficiency and reproducibility of literature identification while maintaining methodological rigor (Lam et al., 2019).

Beyond study selection, AI has also demonstrated utility in data extraction and risk-of-bias assessment. Early investigations employed RobotReviewer, an AI-driven tool designed to automatically extract study characteristics and risk-of-bias items from randomized controlled trials. These applications not only helped standardize the appraisal process but also significantly reduced the workload for human reviewers (Armijo-Olivo et al., 2020; Aali et al., 2020). LLMs can identify selection, performance and detection bias with agreement levels close to human ratings. When paired with structured prompts and the RoB/RoB 2 checklist, these models can assign bias grades and produce visual summaries; in several subtasks their consistency approaches that of human reviewers, although limitations remain regarding training-data bias and the recognition of complex methods. Most authors therefore recommend a dual pathway of AI assistance followed by human verification.

The guideline field provides a useful precedent. A recent scoping review cataloged the progress of generative AI tools in this area and noted that LLMs such as ChatGPT and Claude have already been trialed across multiple tasks—including risk-of-bias assessment—while prototype applications (e.g. “AGREE II Analyzer” and “CPG Risk of Bias”) have been created to enhance objectivity and efficiency (Luo et al., 2024b). Although definitive validation datasets are not yet available, empirical studies in randomized controlled trial and systematic-review appraisal suggest that generative AI can preserve accuracy while substantially accelerating evaluation. The Scientific, Transparent and Applicable Rankings (STAR) working group is now developing a multi-model rating system that integrates several LLM-based tools, further highlighting the considerable potential of generative AI for clinical-practice guideline evaluation (Liu et al., 2024).

In contrast, AI-driven literature quality appraisal is still in its infancy. One analysis of the UK REF2021 dataset predicted scores for 84 966 articles with 72% accuracy by combining random-forest and extreme-gradient boosting algorithms with textual, citation and metadata features (Thelwall et al., 2023). Another investigation based on Nature sub-journals and ICLR proceedings reported that overlap between GPT-4 feedback and expert peer review averaged 30.85% and 39.23%, respectively—similar to inter-expert agreement—and that GPT-4 aligned even more closely on lower-quality papers (43.80%) (Liang et al., 2024a). Overall, evidence in this domain remains scattered, and systematic validation is still limited.

In parallel, scholars have also investigated how LLMs are reshaping broader academic evaluation practices. For example, analyses of peer reviews from leading AI conferences have introduced distributional quantification frameworks to estimate the proportion of reviews substantially modified by LLMs, thereby highlighting their penetration into peer review processes (Liang et al., 2024b). Other studies have proposed collaborative frameworks that integrate human expertise with AI-generated knowledge to assess the innovativeness of academic papers, demonstrating the feasibility of hybrid human–AI models in scholarly evaluation (Wu et al., 2025). These findings underscore both the opportunities and challenges of incorporating LLMs into quality-related academic assessment. However, systematic validation of LLMs' performance in the specific context of literature quality appraisal for systematic reviews and meta-analyses remains to be established.

The contributions of this study are fourfold. First, it extends the application of LLMs from peer-review assistance to literature-quality appraisal in systematic reviews and meta-analyses, focusing on a step that is critical to the reliability of evidence synthesis. Second, it systematically compares AI-generated ratings with human assessments across four widely used appraisal instruments (AMSTAR2, CASP, PEDro, ROB2), providing a comprehensive evaluation of accuracy, consistency, and efficiency. Third, it introduces an entropy-based consistency index as a novel methodological tool, which is more sensitive than conventional concordance statistics in capturing rating stability and divergence. Finally, by validating the approach within the sports-science domain—where these tools are maturely applied—the study demonstrates the practical feasibility of AI-assisted quality appraisal and offers methodological insights for extending its use to other disciplines such as medicine, education, and social sciences.

## 2 Methods

### 2.1 Overview of commonly used literature-quality appraisal tools

Systematic reviews and meta-analyses depend on structured appraisal tools to ensure methodological rigor. In this study, five widely used instruments were considered: PRISMA, AMSTAR 2, CASP, PEDro, and ROB 2.

PRISMA (Preferred Reporting Items for Systematic Reviews and Meta-Analyses) is a reporting guideline with 27 items that promotes transparency and completeness in systematic review reporting. Although indispensable for ensuring standardized reporting, PRISMA does not generate numerical or categorical quality scores. For this reason, it was not included in the subsequent concordance analyses, which required tools capable of producing ratings directly comparable between human reviewers and LLMs (Page et al., 2021; Ardern et al., 2022).

AMSTAR 2 (A MeaSurement Tool to Assess Systematic Reviews, version 2) consists of 16 items, including seven critical domains covering protocol registration, search comprehensiveness, study selection, bias control, and data synthesis. Reviews are categorized as high, moderate, low, or critically low quality (Shea et al., 2017).

CASP (Critical Appraisal Skills Programme) offers checklists for multiple study types (e.g., qualitative studies, RCTs, cohort studies). Each checklist contains 10–12 structured questions guiding judgments about appropriateness and validity. CASP does not produce an aggregate score but emphasizes structured critical reflection (Long et al., 2020).

PEDro (Physiotherapy Evidence Database scale) is an 11-item tool for randomized controlled trials in rehabilitation and exercise sciences, of which 10 contribute to a total score ranging from 0 to 10. It focuses on randomization, blinding, and reporting, and is valued for efficiency in large-scale reviews (Cashin and McAuley, 2020).

ROB 2 (Risk of Bias 2), launched by Cochrane in 2019, evaluates internal bias across five domains using structured signaling questions. It outputs both domain-specific and overall judgments, often visualized with traffic-light plots (Sterne et al., 2019).

Together, these instruments represent complementary perspectives: PRISMA ensures reporting transparency, AMSTAR 2 and CASP evaluate methodological quality, and PEDro and ROB 2 focus on trial-specific internal validity.

### 2.2 Study design

#### 2.2.1 Data sources and screening

To examine the consistency between LLM- and human-based quality appraisal, we selected published systematic reviews and meta-analyses in the field of sports science. This domain was chosen because it has long adopted the appraisal instruments under study (AMSTAR 2, CASP, PEDro, and ROB 2), ensuring transparent, replicable, and well-documented scoring results suitable for benchmarking. Specifically, we drew articles from *Sports Medicine* (impact factor 9.4), the *International Journal of Behavioral Nutrition and Physical Activity* (impact factor 5.5), and the *British Journal of Sports Medicine* (impact factor 16.2), according to the most recent *Journal Citation Reports* (Clarivate, 2025).

**Figure 1:**
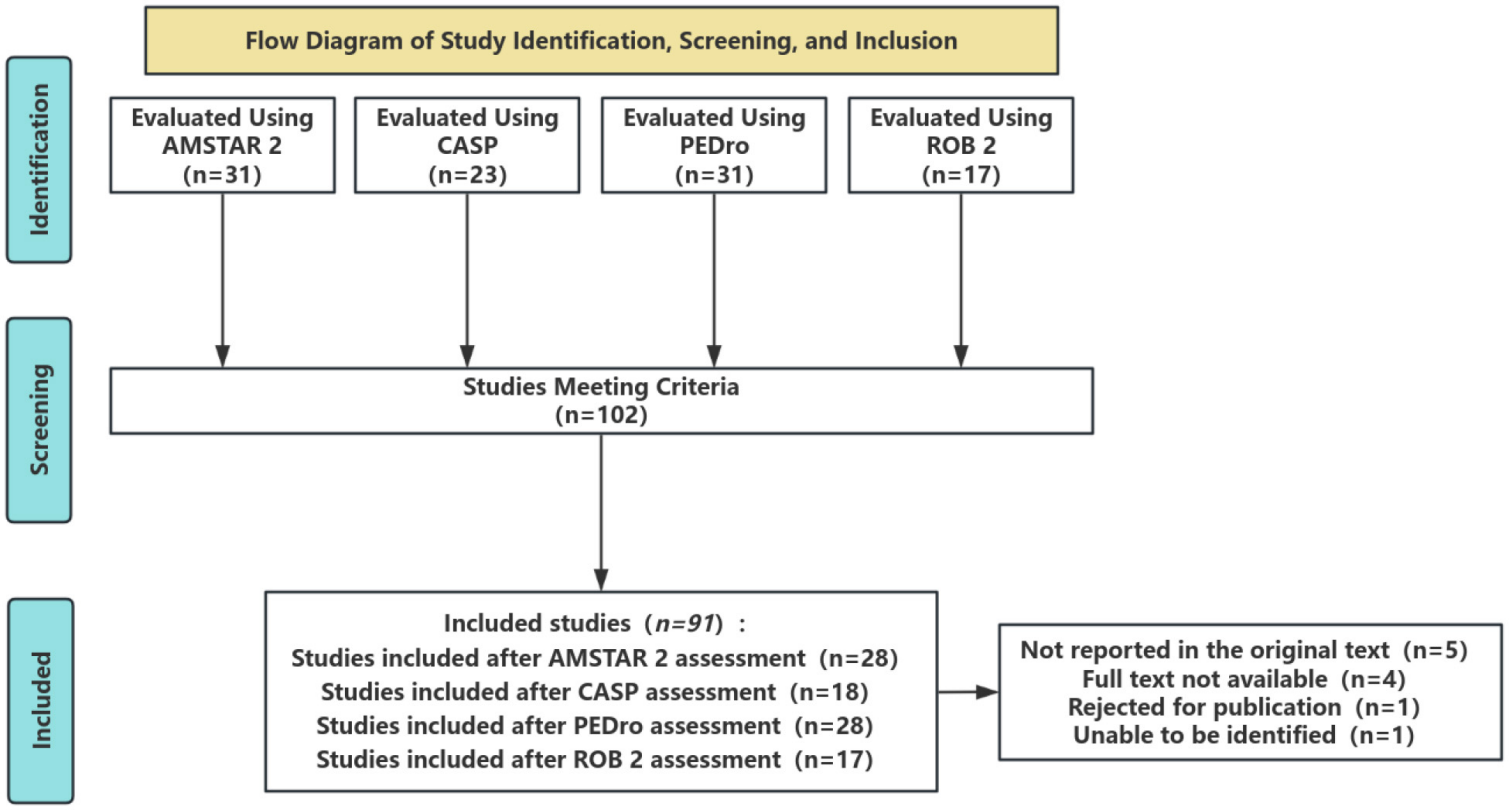
Flow diagram of literature identification, screening and inclusion.

The dataset included 31 AMSTAR 2 assessments of systematic reviews (Gomes-Neto et al., 2024), 23 CASP assessments of primary studies (Peng et al., 2023), 31 PEDro scores of randomized controlled trials (Blagrove et al., 2018), and 17 ROB 2 risk-of-bias evaluations (Milanović et al., 2022). After excluding incomplete records, inaccessible full texts, retracted publications, and metadata-deficient entries, 91 studies remained and were used in the concordance analysis between LLMs and human raters. While the dataset was domain-specific, the selected instruments are widely applied in medicine, education, and social sciences. Therefore, the results are expected to provide insights that are transferable across disciplines, although generalization beyond sports science should be interpreted cautiously. The inclusion process is detailed in Figure 1.

#### 2.2.2 Selection of LLMs

This study selected two representative LLMs: ChatGPT-4o (OpenAI) and DeepSeek R1 (DeepSeek Technology, China). At the time of study implementation, ChatGPT-4o had already accumulated substantial practical applications in evidence-synthesis tasks, demonstrating considerable stability and reliability. All interactions with ChatGPT-4o in this study were conducted via the official ChatGPT application (desktop client), which provided access to the May 2024 release of the model. DeepSeek R1, as a representative open-source model from China, was officially released in January 2025 and was included in this study to enable comparative evaluation across different linguistic and cultural contexts. These two models were chosen because ChatGPT-4o represents the most influential and widely adopted LLM in international research practice, whereas DeepSeek R1 has rapidly become the most downloaded and commonly used LLM in China, far surpassing other domestic and international alternatives. Together, they serve as representative benchmarks for both global and Chinese contexts. Other recently released or emerging models (e.g., GPT-5, Grok, Gemini) had not yet provided sufficiently validated interfaces or stable research environments during the design and implementation of this study and were therefore not included. This choice reflects the objective conditions at the time of study execution rather than methodological limitations.

#### 2.2.3 Prompt design and evaluation protocol

The experiments were conducted on a Microsoft Surface Pro 8 equipped with an 11th-generation Intel Core i5-1145G7 processor (base frequency 2.60 GHz, turbo frequency 2.61 GHz) and 8 GB of memory. All rating procedures were performed under a controlled environment with identical network and power settings to ensure consistency. To simulate the double-blind appraisal process commonly used in traditional practice, each article underwent two independent rounds of scoring: after the model generated an initial result, it was required to conduct a second round without reference to the first, and the second result was recorded as final. In addition, we employed an “expert identity setting” strategy, instructing the LLMs to assume the role of domain specialists proficient in the appraisal tool in use. This design aimed to reflect real-world application scenarios, where end-users are unlikely to provide detailed item-by-item instructions but instead expect AI systems to apply established methodological standards autonomously. The full prompt text used in the experiments is presented in Table 1.

**Table 1:** Prompts used for each literature-quality appraisal tool.

**Tools**	**Prompt**
AMSTAR 2	You are a top-tier literature quality appraisal expert familiar with the AMSTAR 2 (A Measurement Tool to Assess Systematic Reviews) standard. Please evaluate the uploaded article as follows: (1) Evaluation criteria: assess the article item by item in strict accordance with the sixteen AMSTAR 2 items, numbered 1–16. (2) Scoring rules: for every item indicate clearly “Yes”; “No”; “Not Applicable” or “Insufficient Information”. (3) Overall rating: according to the AMSTAR 2 guidance on the number of flaws in critical domains, give an overall quality level of “High”; “Moderate”; “Low” or “Critically low”, and explain the basis for the judgement. Ensure that the appraisal follows the official AMSTAR 2 guidance and shows professionalism and rigor.
CASP	You are a top-tier literature quality appraisal expert familiar with the CASP (Critical Appraisal Skills Programme) standard. Please carry out a systematic evaluation as follows: (1) Study-type confirmation: identify the study design of the article, for example systematic review, qualitative study or randomized controlled trial, and apply the matching CASP framework. (2) Item-by-item scoring: for each item award 1 point if it fully meets the criterion, 0 points if it is unclear or ambiguous, and X if the information is missing. (3) Total score: add the scores of all items to obtain the total score. Follow scientific, fair and rigorous principles, provide sufficient justification and ensure that the results accord with the CASP appraisal logic.
PEDro	You are a top-tier literature quality appraisal expert familiar with the PEDro (Physiotherapy Evidence Database) scale. Please score the article as follows: (1) Study-type confirmation: confirm that the article is a randomized controlled trial because PEDro applies only to RCTs. (2) Item-by-item scoring: the scale has eleven items. Item 1 is not counted in the total score. Items 2 to 11 each score 1 or 0. The maximum total score is 10. (3) Quality grading: based on the total score grade the article as “9–10 Excellent”; “6–8 Good”; “4–5 Fair” or “below 4 Poor”. Judge each item strictly according to the official PEDro standard and present clear, verifiable results.
ROB 2	You are a top-tier literature quality appraisal expert familiar with the ROB 2 (Risk of Bias 2) tool. Please assess the risk of bias in the randomized controlled trial as follows: (1) Study-type confirmation: confirm that the article is an RCT because ROB 2 applies only to RCTs. (2) Item-by-item scoring: analyse the article according to the five ROB 2 bias domains. (3) Scoring options: for each domain choose “Low risk of bias”; “Some concerns”; or “High risk of bias”. (4) Overall judgement: derive the overall bias level from the five domain scores according to ROB 2 guidance. Ensure that the evaluation process is rigorous and transparent, with clear justification that meets the official ROB 2 requirements.

#### 2.2.4 Consistency measurement using entropy-based index

To measure agreement between the two LLMs (ChatGPT-4.0 and DeepSeek R1) and human reviewers, we applied the Entropy-based Consistency Index. Originating from Shannon's information theory (Shannon, 1948), this method is widely used in artificial-intelligence evaluation, information engineering and complex-system modeling because it quantifies the concentration and stability of ratings provided by multiple assessors. Compared with traditional concordance statistics, the entropy approach remains stable when rating scales differ or subjective bias is present. A recent investigation showed that entropy-based measures outperform conventional indices when assessing model generalization and extracting information structure (Guha and Velegol, 2023).

For each article, we first calculated the item-level entropy and then the overall entropy from the distribution of rating categories. Consistency levels were assigned according to these entropy values. The procedure is outlined below.

Let an article contain *n* appraisal items. Each item is rated by three assessors (human reviewer, ChatGPT-4.0, DeepSeek R1) using *k* possible rating categories. For the *i*-th item, let *P*_*ij*_ denote the relative frequency with which the *j*-th category ( *j* = 1, 2, …,*k* ) is chosen by the three assessors, such that:


$$\begin{array}{l} \overset{k}{\underset{j=1}{\sum }}P_{ij}=1,\;P_{ij}\geq 0\; \end{array}$$ (1)

The entropy for the *i*-th appraisal item is calculated as:


$$\begin{array}{l} H_{i}=-\overset{k}{\underset{j=1}{\sum }}P_{ij}\log_{2}P_{ij} \end{array}$$ (2)

Where *H*_*i*_ represents the consistency entropy of the *i*-th rating item and is used to measure the degree of agreement among the three raters for that item. A smaller *H*_*i*_ indicates a more concentrated distribution of ratings and thus stronger agreement, whereas a larger *H*_*i*_ signals a more dispersed distribution and weaker agreement.

To evaluate the overall agreement of each article across all appraisal items, we calculated the mean entropy (*H*_*mean*_), defined as the arithmetic average of the item-level entropies:


$$\begin{array}{l} H_{mean}=\frac{1}{n}\overset{n}{\underset{i=1}{\sum }}H_{i} \end{array}$$ (3)

where *H*_*mean*_ serves as a composite indicator of rating consistency and enables comparisons across articles and appraisal tools. The theoretical upper bound of entropy is *log*_2_*k*, which represents maximum uncertainty when all *k* rating categories are equally likely. Entropy values are expressed in bits. Both item-level and mean entropies were graded according to empirical cut-offs, as summarized in Table 2.

**Table 2:** Entropy-based grading of rating consistency.

**Entropy (bits)**	**Consistency level**
0	Perfect agreement
(0,0.3]	Very high
(0.3,0.5]	High
(0.5,0.7]	Moderately high
(0.7,1.0]	Moderate
>1.0	Low

In addition, the generation time for each article was recorded for both models, and a two-round timing protocol was used to minimize the influence of network latency and model variability. Because neither rating time nor entropy values satisfied the assumption of normality (Shapiro–Wilk test, *p* < 0.001), the relationship between timing variability and consistency was analyzed with Spearman's rank correlation.

## 3 Results

### 3.1 Overall consistency across appraisal tools

Entropy-based analysis of the 91 included articles showed that quality ratings generated by ChatGPT-4.0 and DeepSeek R1 matched human assessments closely, with a mean global entropy of 0.422—classified as “high” (Table 3). Agreement levels varied by appraisal tool: AMSTAR 2 produced a global entropy of 0.700 (“moderately high”), while CASP and PEDro yielded lower values of 0.252 and 0.167, respectively, both corresponding to “very high”. In contrast, ROB 2 returned an entropy of 0.570, again within the “moderately high ” range. Taken together, these results indicate that the large-language models aligned particularly well with manual reviewers when the scoring scheme was straightforward (CASP, PEDro), whereas tools that require multiple judgement steps (AMSTAR 2, ROB 2) generated slightly higher entropy values.

**Table 3:** Entropy-based agreement across appraisal instruments (*n* = 91).

**Tools**	**Minimum**	**Maximum**	**SD**	**Mean**	**Consistency level**
AMSTAR 2	0.432	1.046	0.137	0.700	Moderately high
CASP	0.092	0.592	0.130	0.252	Very high
PEDro	0.077	0.230	0.036	0.167	Very high
ROB 2	0.153	0.987	0.230	0.570	Moderately high
Overall	0.077	1.046	0.133	0.422	High

### 3.2 Consistency by appraisal tools

**Figure 2:**
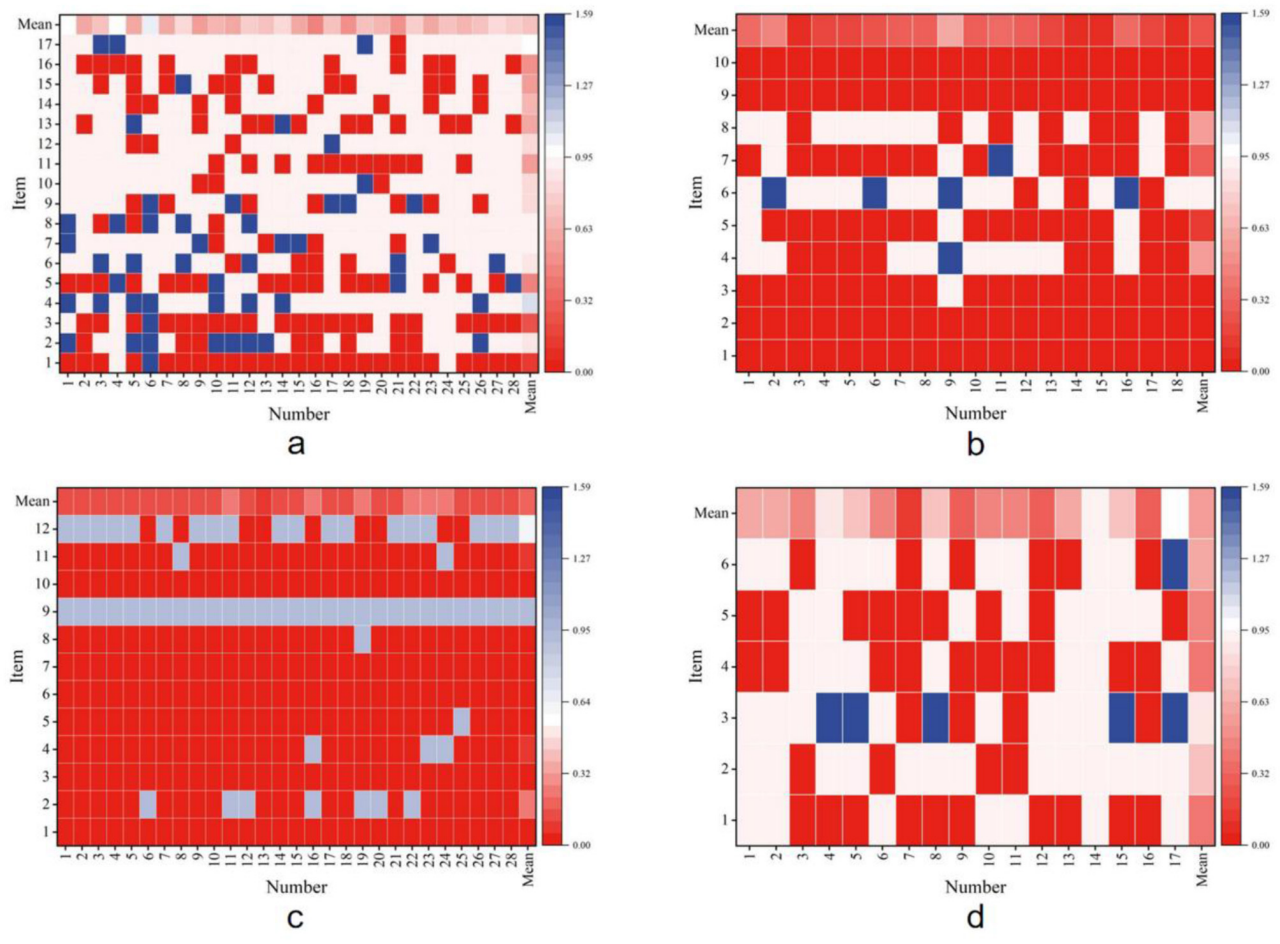
Entropy heatmaps of consistency across four literature-quality appraisal tools. **(a)** AMSTAR 2, **(b)** CASP, **(c)** PEDro, **(d)** ROB 2.

Entropy-based agreement varied substantially across the four appraisal instruments (Figures 2a–d), reflecting differences in tool structure and scoring logic.

For AMSTAR 2 (*n* = 28), the mean global entropy was 0.700, corresponding to “moderately high consistency.” Study-level results showed considerable heterogeneity: one review (3.6%) achieved “high consistency,” 13 reviews (46.4%) fell into the “moderately high” range, another 13 (46.4%) were rated “moderate,” and one review (3.6%) was classified as “low.” At the item level, agreement was strongest for Items 1 and 3 (“very high”), moderate for Items 2, 6–10 and 12 (“moderate”), and weakest for Item 4 (“low”). Five items—including protocol registration and synthesis methods—reached the “moderately high” band, illustrating the challenge of applying subjective multi-step judgements across domains.

In contrast, CASP (*n* = 18) demonstrated consistently stronger agreement. The mean entropy was 0.252, classified as “very high consistency.” Fourteen studies (77.8%) achieved “very high” and the remaining four (22.2%) were in the “high” range, with no studies falling below this threshold. At the item level, perfect agreement was observed for Items 1, 2, 9, and 10, while Items 3, 5 and 7 were rated “very high.” Only Item 6 showed “moderate” consistency. These results suggest that the binary and logic-guided question format of CASP made it easier for LLMs to align with human reviewers.

The PEDro scale (*n* = 28) yielded the strongest alignment of all instruments. The mean entropy was only 0.167, firmly within the “very high consistency” category. Every included study was rated in this band. Item-level analysis revealed five items (41.7%) with perfect agreement (Items 1^*^, 3, 6, 7, 10) and another five (41.7%) with “very high” consistency. Only two items—Items 9 and 12—deviated, at “moderate” and “moderately high,” respectively. The clear rules of PEDro, combined with its numerical scoring system, appear particularly well-suited to LLM processing, leaving little room for interpretive ambiguity.

By comparison, ROB 2 (*n* = 17) produced more mixed outcomes. The mean entropy was 0.570, corresponding to “moderately high consistency.” At the study level, one review (5.9%) reached “very high,” seven (41.2%) were rated “high,” three (17.6%) “moderately high,” and six (35.3%) “moderate.” Item-level distribution was similarly varied: Items 1, 4 and 8 were rated “high,” Item 6 was “moderately high,” while Items 2 and 3 achieved only “moderate” agreement. This variability reflects the tool's reliance on domain-specific signaling questions and nuanced bias judgments, which present greater challenges for LLMs interpretation.

### 3.3 Time-cost analysis of quality appraisal

#### 3.3.1 Processing-time differences between LLMs and manual review

The two LLMs completed quality ratings substantially faster than human reviewers. Across all four appraisal tools, ChatGPT-4.0 required a mean of 19.19 s per article, whereas DeepSeek R1 averaged 52.28 s, yielding a combined mean of 33.09 s. Published benchmarks for manual appraisal are considerably longer: approximately 20 min for a full AMSTAR 2 assessment (De Santis and Matthias, 2023), 10–15 min for PEDro (Physiotherapy Evidence Database, 2025), 30 min for RoB 2 (Richter and Hemmingsen, 2025), and 30–60 min for a 10-item qualitative checklist analogous to CASP (Duval et al., 2023). Overall, the LLMs were about 47.8 times faster than the human appraisal workflow. A detailed comparison is provided in Table 4.

**Table 4:** Per-article appraisal time for ChatGPT-4.0 and DeepSeek R1 compared with manual evaluation (seconds).

**Tools**	**Models**	**Minimum**	**Maximum**	**Mean**	**95%CI**
AMSTAR2	ChatGPT-4.0	12.83	24.00	16.94	15.98–17.99
	DeepSeek R1	31.28	111.69	65.02	56.25–73.78
	Manual	–	–	1,200.00	–
CASP	ChatGPT-4.0	18.08	28.21	22.26	20.98–23.55
	DeepSeek R1	28.97	157.97	58.22	43.91–72.52
	Manual	–	–	750.00	–
PEDro	ChatGPT-4.0	13.41	25.32	18.41	17.16–19.66
	DeepSeek R1	31.83	64.26	44.95	41.66–48.25
	Manual	–	–	2,700.00	–
ROB2	ChatGPT-4.0	15.16	25.63	19.13	17.56–20.70
	DeepSeek R1	29.83	61.57	40.92	36.62–45.23
	Manual	–	–	1,680.00	–
Overall	ChatGPT-4.0	12.83	28.21	19.19	18.16–19.59
	DeepSeek R1	28.97	157.97	52.28	48.50–57.23
	Manual	–	–	1,582.50	–

#### 3.3.2 Between-model processing time

**Figure 3:**
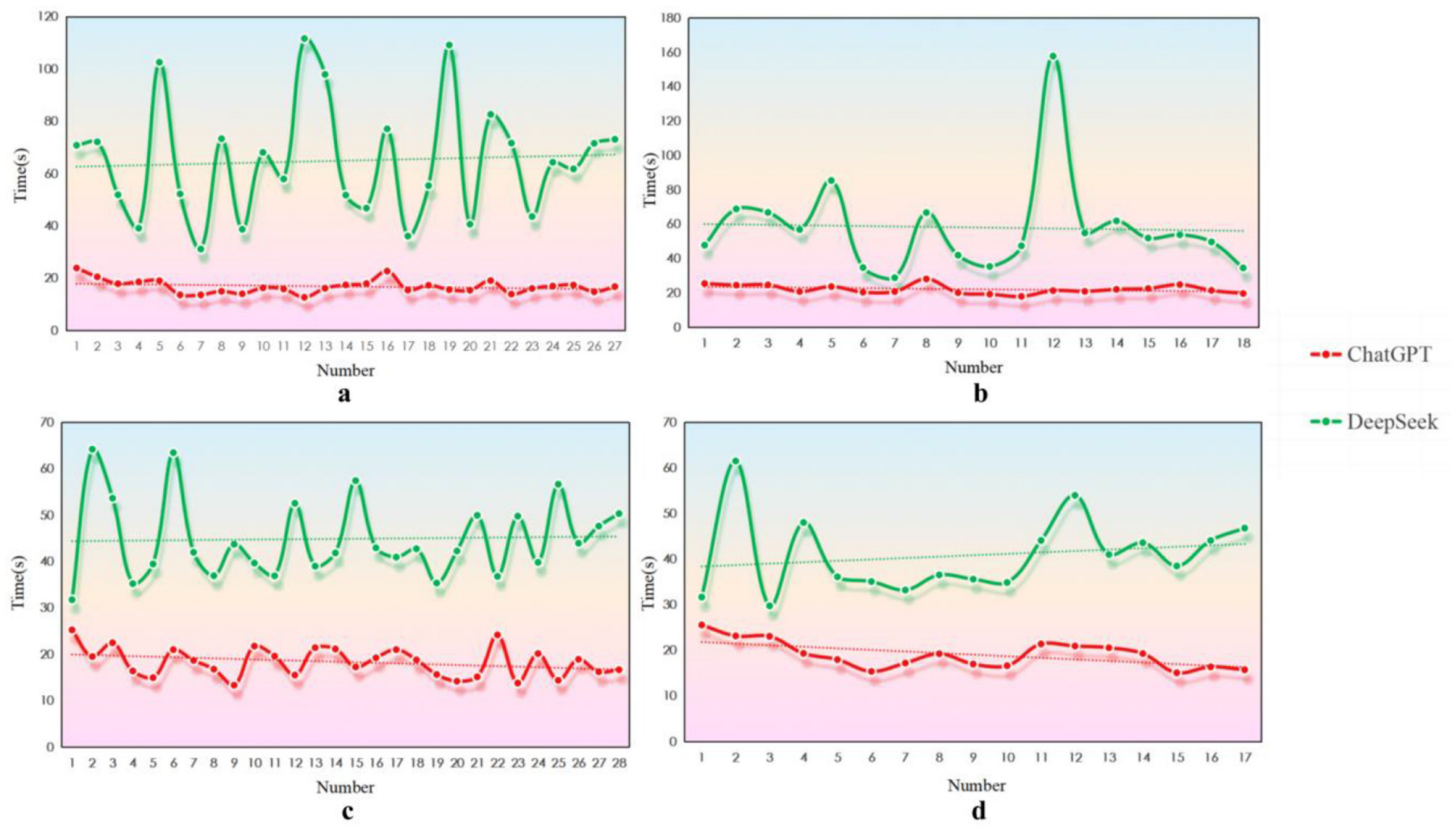
Processing-time differences between ChatGPT-4.0 and DeepSeek R1 during literature-quality appraisal. **(a)** AMSTAR 2, **(b)** CASP, **(c)** PEDro, **(d)** ROB 2.

Across all four appraisal instruments, ChatGPT-4.0 consistently completed ratings more rapidly than DeepSeek R1. On AMSTAR 2, the mean appraisal times were 16.9 s for ChatGPT-4.0 and 65.0 s for DeepSeek R1, representing an additional 48.1 s for the latter. For CASP, the models averaged 22.3 and 58.2 s, respectively, with DeepSeek R1 requiring 35.9 s more. PEDro ratings took 18.4 s with ChatGPT-4.0 vs. 45.0 s with DeepSeek R1, a difference of 26.5 s. For RoB 2 assessments, ChatGPT-4.0 averaged 19.1 s compared with 40.9 s for DeepSeek R1, extending the time by 21.8 s. When pooled across instruments, DeepSeek R1 required an average of 33.1 s more per article. The gap between models ranged from 6.1 to 136.5 s for individual studies. Figures 3a–d illustrates these differences across appraisal tools.

#### 3.3.3 Relation between processing-time variability and rating consistency

**Figure 4:**
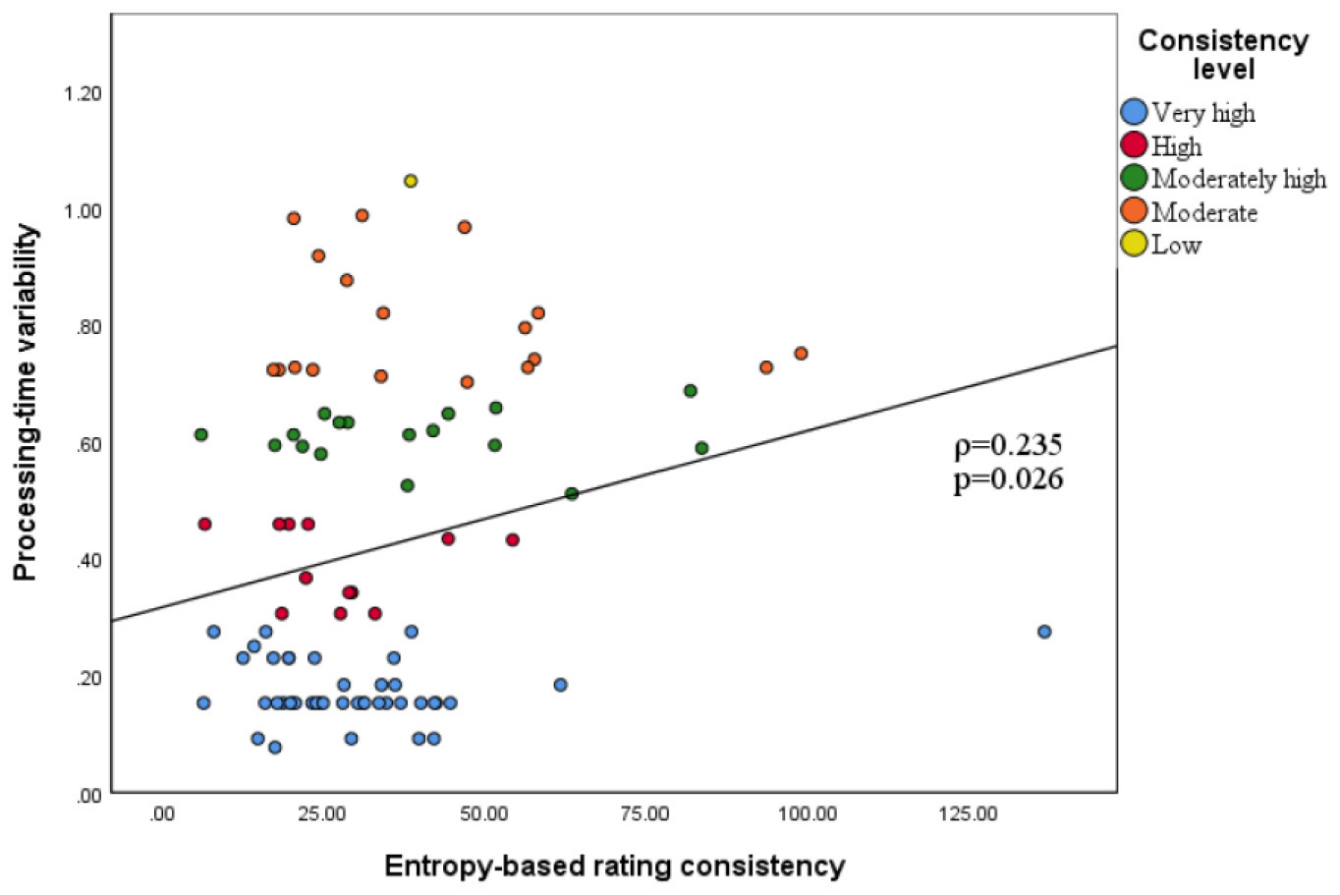
Relation between processing-time variability and entropy-based rating consistency.

Spearman's rank-order analysis revealed a modest yet statistically significant positive association between processing-time variability and entropy (ρ = 0.235, *p* = 0.026). Hence, greater fluctuations in generation time were associated with lower agreement, as reflected by higher entropy values. As illustrated in Figure 4, articles that achieved “very high consistency” clustered in the region of minimal time variation, whereas the proportion of lower-consistency cases increased with greater variability. The coefficient of determination was low (*R*^2^ = 0.055), indicating that processing-time instability explained only a small proportion of the variance in entropy and is unlikely to represent the main determinant of rating disagreement.

**Figure 5:**
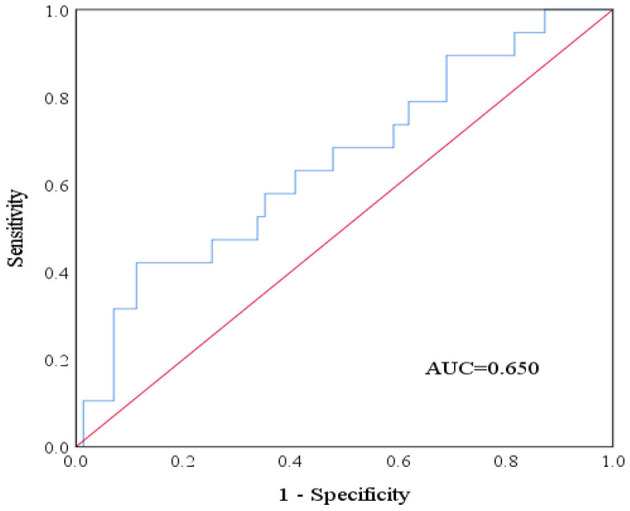
ROC Curve between processing-time variability and entropy-based rating consistency.

To establish an operational threshold for practical application, we defined items with moderate or lower rating consistency (entropy > 0.7) as the target condition and performed a receiver operating characteristic (ROC) curve analysis using processing-time variability as the test variable. Figure 5 illustrates that the area under the curve (AUC) was 0.650 (*p* = 0.045), indicating a moderate level of discriminative performance. The optimal cutoff point, determined by the maximum Youden index (Se + Sp – 1), was 45.795 s (rounded to 46 s), with a sensitivity of 0.421 and a specificity of 0.887. Thus, when the model's processing-time variability for a single article exceeded 46 s, the probability of producing a rating with moderate or lower consistency increased significantly. This threshold may serve as a practical reference for initiating manual review in AI-assisted appraisal workflows.

## 4 Discussion

The ability to use AI in a reasoned, efficient and systematic manner is increasingly regarded as a hallmark of methodological competence. In an era of data saturation, improving the speed, accuracy and reproducibility of evidence synthesis has become central to research practice. The present study addressed two long-standing barriers in literature appraisal—time cost and subjectivity—by evaluating the performance of ChatGPT-4.0 and DeepSeek R1 across four widely adopted instruments (AMSTAR2, CASP, PEDro and ROB2). Using an entropy-based consistency index derived from information theory, we quantified agreement between the two LLMs and human reviewers. Both models achieved high overall concordance with manual ratings (mean entropy 0.422), and for instruments with well-defined decision anchors such as CASP (0.252) and PEDro (0.167), agreement reached the “very high” category. These findings demonstrate that LLMs can already generate standardized quality ratings that approximate expert judgement while operating at a fundamentally different speed scale.

### 4.1 Practical advantages of LLM-assisted appraisal

Compared with manual evaluation, LLMs demonstrated four significant advantages in this study, each of which highlights their potential to reshape the workflow of evidence synthesis.

First, LLMs markedly reduced appraisal time. Traditional manual quality assessment typically requires 20–30 min per article, and even experienced reviewers spend an average of *M* = *16.2, SD* = *4.7 min* applying RoB 2 to a single study (Duval et al., 2023). In more complex cases, a full risk-of-bias assessment may take nearly 6 h per study (Glujovsky and Ciapponi, 2023). By contrast, ChatGPT-4.0 and DeepSeek R1 required only 19.2 and 52.3 s per article, respectively, representing an approximately 49-fold acceleration. This substantial improvement in efficiency not only shortens the timeline for systematic reviews, meta-analyses, and guideline development but also reduces the human resources required in academic and clinical contexts.

Second, AI ratings exhibited high transparency. While DeepSeek R1 required slightly more time than ChatGPT-4.0, it provided detailed item-level scores along with explicit reasoning, supporting text evidence, and concise summaries of study strengths and limitations. Such explainable outputs, which typically demand additional effort in manual reviews, were generated instantly by the model. ChatGPT-4.0, by contrast, produced a more compact report but remained stable in both speed and accuracy, making it more suitable for time-sensitive or large-scale tasks. These complementary features suggest that different models can be strategically selected based on task requirements.

Third, LLMs enable efficient large-scale parallel processing. Manual workflows generally rely on two independent reviewers plus a third for adjudication, a time-consuming and labor-intensive process. This inefficiency is particularly evident in large-scale evidence syntheses where the per-article time commitment increases substantially. LLMs, however, can analyze dozens of papers in parallel within seconds, making them particularly well suited for initial screening, rapid batch appraisal, and quality control within systematic reviews and meta-analyses. Given the exponential growth of scientific publications, this capability will become increasingly indispensable.

Finally, standardized reasoning reduces subjectivity. Human evaluations are influenced by reviewer expertise, interpretation, and even fatigue, leading to inter-rater variability and intra-rater inconsistency upon reassessment. For instance, ROB 2 assessments often yield divergent results across reviewer groups, necessitating time-consuming consensus meetings (Glujovsky and Ciapponi, 2023). In contrast, LLMs guided by fixed prompts follow consistent reasoning pathways and retrieval strategies, producing outputs that are more reproducible and objective. This not only enhances rating stability but also paves the way for standardized and auditable workflows in future evidence synthesis.

### 4.2 Variation across appraisal instruments

The differences in consistency across appraisal tools were substantial, reflecting three main dimensions: the clarity of item semantics, the breadth of information extraction, and the complexity of reasoning chains. PEDro, designed specifically for randomized controlled trials, consists primarily of binary items (Yes/No) with highly explicit wording, enabling the models to directly map textual evidence to evaluation outcomes. Because the required information is usually presented in a straightforward manner, both models were able to rapidly identify key details, resulting in near-perfect agreement. Similarly, CASP adopts a question–answer framework with concise items, short reasoning paths, and unambiguous language, allowing AI models to complete judgments without cross-paragraph synthesis. This design also produced very high consistency.

In contrast, AMSTAR 2 and ROB 2 posed far greater challenges. These tools not only address risk-of-bias assessment but also demand integrated evaluations across multiple methodological dimensions, such as the comprehensiveness of the search strategy, dual independence in study selection and data extraction, and the potential influence of missing data on study outcomes. Such information is often dispersed across different sections of a paper (introduction, methods, and results) and may require causal chain reasoning within a broader research context. For current LLMs, limitations in long-context integration and causal inference remain well documented; therefore, higher entropy values and lower consistency observed in AMSTAR 2 and ROB 2 are expected outcomes.

Further analysis indicates that the degree of clarity and objectivity of individual items is a key determinant of consistency. Explicit and objective items are more likely to achieve high agreement. For instance, PEDro items on randomization and blinding are generally reported explicitly, enabling both models to deliver nearly perfect alignment with human reviewers. CASP items such as “Was there a clear statement of the research aims?” and “Was there a clear statement of the findings?” also fall into this category, and all achieved perfect agreement in the present analysis, demonstrating that models perform reliably when facing semantically direct, short reasoning tasks.

By contrast, items requiring implicit reasoning and cross-paragraph synthesis posed greater difficulties. For example, the AMSTAR 2 item “Did the review authors use a comprehensive literature search strategy?” requires synthesizing evidence on database selection, time ranges, keyword design, and inclusion of gray literature—factors often distributed unevenly and reported inconsistently. Similarly, the ROB 2 domain of “bias due to deviations from intended interventions” is a prototypical complex item, requiring an understanding of study design intentions as well as interpretation of how deviations might affect outcomes. These items lack a single decisive anchor, and even human reviewers often disagree in their assessments. Unsurprisingly, LLMs were more prone to rating drift and lower agreement on such tasks.

### 4.3 Theoretical and methodological implications

The findings indicate that rating consistency depends not only on the complexity of individual items but also on the model's ability to interpret prompts, construct scoring logic, and integrate contextual information. Although ChatGPT-4.0 and DeepSeek R1 performed stably in most situations, both models displayed rigid, templated characteristics when faced with tasks requiring subjective interpretation, multi-step reasoning, or ambiguous semantics. For example, in Item 9 of the PEDro scale, ChatGPT-4.0 withheld a point unless the use of an intention-to-treat (ITT) analysis was explicitly stated, while DeepSeek R1 awarded the point if no participants were lost to follow-up, interpreting complete data as equivalent to ITT; in contrast, human reviewers credited every study. This discrepancy did not arise from information retrieval failure but from divergent interpretations of the scoring rules, underscoring the need for LLMs to develop greater flexibility when multiple valid reasoning paths exist.

From a theoretical perspective, this study highlights the “double-edged” nature of LLMs in evidence-based research. On the one hand, LLMs demonstrate highly stable and reproducible judgments on structured and rule-based items, often approaching or even surpassing human performance in terms of speed and consistency. On the other hand, they continue to face limitations in handling vague semantics, long reasoning chains, or tasks requiring cross-disciplinary expertise. These results provide empirical evidence for understanding the boundaries of LLM reasoning and offer a novel lens through which to examine the integration of AI into evidence-based science.

At the methodological level, the findings suggest that the optimal future pathway may be a hybrid workflow of “AI prescreening plus human verification.” In this model, LLMs can take responsibility for large-scale, standardized, and repetitive tasks, leveraging their efficiency and consistency to reduce labor demands. Meanwhile, complex or high-stakes items requiring integrated judgment would remain under expert human review to ensure rigor. Such a workflow balances efficiency with reliability and provides a feasible framework for quality control in systematic reviews and meta-analyses.

Moreover, this study offers methodological insights for developing new appraisal systems. By introducing an entropy-based consistency index, we extended the application boundaries of traditional agreement metrics and provided a quantitative foundation for an AI-led independent appraisal framework. This methodological innovation demonstrates that LLMs need not be confined to a “human replacement” role; instead, they can help drive the evolution of evidence-based research methods by enabling new modes of evaluation. In broader terms, this cross-disciplinary approach—integrating AI, statistics, and evidence-based science—may provide transferable insights for quality appraisal research in medicine, education, and the social sciences. We also acknowledge that the relatively generic prompt phrasing adopted in this study may have limited the models' ability to fully accommodate the nuanced requirements of more complex instruments such as AMSTAR 2 and RoB 2. However, this design choice was deliberate, aiming to ensure comparability across tools and to approximate realistic usage scenarios for non-specialist users. Future research could build on this foundation by exploring more targeted, tool-specific prompt refinements and by directly comparing two input modes (“paper + generic prompts” vs. “paper + complete evaluation criteria”) to examine their effects on consistency, discriminative performance, processing time, and error patterns, thereby further enhancing model performance in complex appraisal tasks.

### 4.4 Limitations

This study has several limitations that should be considered when interpreting the findings. First, the dataset consisted of 91 articles in sports and exercise science, all written in English and largely presented in structured formats. While the relative maturity of quality appraisal practices in this field ensured a degree of consistency, it also constrained the external validity of our results. The exclusion of non-English literature, gray literature, and unstructured reports may underestimate the challenges faced by LLMs in more complex and diverse contexts, thereby limiting the generalizability of the conclusions across disciplines. Second, the model selection was restricted to ChatGPT-4.0 and DeepSeek R1. At the time of study implementation, ChatGPT-4.0 represented one of the most stable and widely validated international systems for evidence-based research tasks, while DeepSeek R1 provided a representative open-source option within the Chinese context. However, newer and widely used models such as GPT-5, Grok, and Gemini were not included, narrowing the scope of comparison. Future studies should broaden model coverage to enable more comprehensive benchmarking. Third, human ratings were adopted as the reference standard, though they are not infallible. For example, the discrepancies observed in the PEDro ITT item likely reflected equally valid yet different interpretations rather than model error. Fourth, during batch operations both models occasionally produced incomplete outputs or minor computational errors, highlighting the rigidity of fixed-prompt frameworks and the limited adaptability of LLMs when processing non-standardized or structurally complex texts. Finally, this study focused primarily on score concordance and efficiency. It did not explore how factors such as model architecture, parameter size, or prompt strategy influence performance, leaving the underlying mechanisms of discrepancy unresolved.

## 5 Conclusion and future work

This study provides empirical evidence that LLMs, specifically ChatGPT-4.0 and DeepSeek R1, can markedly accelerate literature quality appraisal while maintaining high levels of agreement with human reviewers. The mean processing time decreased from 1,582.50 s per article with manual evaluation to only 33.09 s with LLMs—an approximately 48-fold improvement in efficiency. Importantly, this gain in speed was not accompanied by a reduction in accuracy. The global mean entropy was 0.42, corresponding to a “high” level of consistency. Agreement was strongest for instruments with binary or rule-based scoring structures, such as PEDro (0.17) and CASP (0.25), both of which reached the “very high” category. By contrast, entropy values were higher for AMSTAR 2 (0.70) and RoB 2 (0.57), reflecting the greater complexity of multi-domain judgments that require long-context integration and subjective interpretation. Item-level analyses further indicated that discrepancies typically arose from differences in interpreting scoring rules—such as the application of intention-to-treat (ITT) criteria—rather than from failures in information retrieval. For more complex tasks, longer and more variable processing times tended to be associated with lower consistency, suggesting that monitoring generation time could serve as a practical indicator to trigger manual verification. Overall, these findings demonstrate that AI-assisted appraisal based on LLMs offers significant advantages in efficiency, reproducibility, and transparency, positioning such tools as a viable complement—and in some contexts, a potential alternative—to manual review. As models continue to advance, they are expected not only to exceed human raters in speed but also to approach, and potentially surpass, them in accuracy, provided that task-specific adaptations are implemented.

Looking forward, several directions for future work emerge from this study. First, a hybrid workflow combining AI prescreening with human verification should be implemented: LLMs can manage large-scale factual checks and structured items, while domain experts focus on nuanced or interpretive judgments. Second, for appraisal items with lower agreement, refined prompt engineering and domain-specific fine-tuning should be pursued to enhance contextual comprehension, logical reasoning, and causal inference. Stepwise prompting and iterative verification strategies may further improve robustness in complex scenarios. Third, future research should explore the development of domain-embedded LLMs that integrate sport-science terminology, expert heuristics, and explicit appraisal rules, thereby improving their utility in specialized fields such as kinesiology, rehabilitation, and sports medicine. Finally, given the limitations of human ratings as a “reference standard,” we advocate for the construction of an independent AI-led appraisal framework capable of flagging methodological weaknesses that may be overlooked in high-scoring human assessments. Such a framework could enhance the objectivity, scalability, and screening power of evidence synthesis, and ultimately contribute to more rigorous and transparent knowledge production.

## Data Availability

The original contributions presented in the study are included in the article/Supplementary material, further inquiries can be directed to the corresponding author.
