# Supplementary material for: Can AI assess literature like experts? An entropy-based comparison of ChatGPT-4o, DeepSeek R1, and human ratings
Source: Front Res Metr Anal. 2025 Nov 10;10:1684137. doi: 10.3389/frma.2025.1684137 (PMC12640921; doi:10.3389/frma.2025.1684137)
Supplement: Supplementary file 1 [file Table_1.docx]

Suppl.1 Results of large language models and human reviewers across different tools

Table 1 Results of large language models and human reviewers in AMSTAR 2

| References | Tools | Item 1 | Item 2 | Item 3 | Item 4 | Item 5 | Item 6 | Item 7 | Item 8 | Item 9 | Item 10 | Item 11 | Item 12 | Item 13 | Item 14 | Item 15 | Item 16 | Quality |
| --- | --- | --- | --- | --- | --- | --- | --- | --- | --- | --- | --- | --- | --- | --- | --- | --- | --- | --- |
| Bourscheid et al. 2021 | Human | Yes | PY | Yes | PY | Yes | Yes | No | PY | PY | No | No | No | No | No | No | Yes | Critically low |
|  | ChatGPT4.0 | Yes | NA | No | No | Yes | Yes | Yes | INS | No | No | Yes | NA | INS | Yes | Yes | Yes | Low |
|  | DeepSeekR1 | Yes | Yes | Yes | Yes | Yes | No | Yes | Yes | No | Yes | Yes | No | No | No | No | No | Low |
| Chen & Tang 2021 | Human | Yes | No | Yes | PY | No | No | No | PY | Yes | No | No | No | No | No | Yes | Yes | Critically low |
|  | ChatGPT4.0 | Yes | No | Yes | Yes | No | Yes | Yes | Yes | Yes | No | Yes | No | No | Yes | No | Yes | Critically low |
|  | DeepSeekR1 | Yes | No | Yes | Yes | No | No | Yes | Yes | No | Yes | Yes | Yes | No | Yes | No | Yes | Moderate |
| Chen et al. 2016 | Human | Yes | No | Yes | PY | Yes | No | No | Yes | PY | No | No | No | No | No | Yes | Yes | Critically low |
|  | ChatGPT4.0 | Yes | No | Yes | Yes | Yes | Yes | No | Yes | Yes | No | Yes | NA | INS | Yes | Yes | Yes | Low |
|  | DeepSeekR1 | Yes | INS | Yes | No | Yes | INS | Yes | Yes | INS | INS | Yes | Yes | No | Yes | Yes | Yes | Moderate |
| Cugusi et al. 2020 | Human | No | PY | Yes | PY | No | No | No | No | No | No | No | No | No | No | No | Yes | Critically low |
|  | ChatGPT4.0 | No | Yes | NA | No | INS | INS | No | INS | Yes | Yes | Yes | Yes | Yes | Yes | INS | Yes | Low |
|  | DeepSeekR1 | Yes | Yes | Yes | PY | Yes | No | Yes | Yes | No | No | Yes | No | Yes | Yes | No | Yes | Moderate |
| Du et al. 2021 | Human | Yes | PY | Yes | No | Yes | Yes | No | Yes | Yes | No | No | No | Yes | Yes | Yes | Yes | Critically low |
|  | ChatGPT4.0 | Yes | No | Yes | INS | Yes | NA | No | Yes | Yes | INS | No | No | No | Yes | Yes | Yes | Critically low |
|  | DeepSeekR1 | Yes | Yes | Yes | Yes | Yes | INS | Yes | Yes | Yes | No | Yes | No | INS | Yes | Yes | Yes | Moderate |
| Elliott et al. 2015 | Human | Yes | No | No | PY | No | No | No | PY | Yes | No | No | No | No | No | No | No | Critically low |
|  | ChatGPT4.0 | INS | No | Yes | Yes | Yes | Yes | No | No | INS | Yes | Yes | No | No | No | No | Yes | Critically low |
|  | DeepSeekR1 | No | Yes | INS | No | No | No | Yes | Yes | No | Yes | No | No | Yes | No | Yes | Yes | low |
| Fan et al. 2021 | Human | Yes | PY | Yes | PY | Yes | Yes | No | PY | Yes | No | No | No | No | Yes | Yes | Yes | Critically low |
|  | ChatGPT4.0 | Yes | Yes | Yes | Yes | Yes | Yes | No | Yes | Yes | Yes | Yes | Yes | Yes | No | Yes | Yes | Moderate |
|  | DeepSeekR1 | Yes | Yes | Yes | Yes | Yes | INS | Yes | Yes | Yes | No | Yes | Yes | Yes | No | Yes | Yes | Moderate |
| Garcia et al. 2019 | Human | Yes | No | Yes | No | Yes | No | No | PY | Yes | No | No | No | No | No | Yes | Yes | Critically low |
|  | ChatGPT4.0 | Yes | No | Yes | No | Yes | INS | Yes | No | Yes | Yes | No | No | No | Yes | INS | Yes | Critically Low |
|  | DeepSeekR1 | Yes | No | Yes | Yes | Yes | Yes | Yes | Yes | No | Yes | Yes | Yes | Yes | Yes | No | No | Low |
| Gomes-Neto et al. 2017 | Human | Yes | No | Yes | No | Yes | Yes | No | PY | PY | No | No | No | No | Yes | No | Yes | Critically low |
|  | ChatGPT4.0 | Yes | No | Yes | Yes | Yes | Yes | INS | Yes | No | No | Yes | NA | NA | Yes | Yes | Yes | Critically low |
|  | DeepSeekR1 | Yes | No | Yes | Yes | Yes | No | Yes | Yes | No | No | Yes | Yes | No | Yes | No | No | low |
| Hannan et al. 2018 | Human | Yes | PY | Yes | PY | No | Yes | No | Yes | PY | No | Yes | No | No | Yes | Yes | Yes | Critically low |
|  | ChatGPT4.0 | Yes | No | Yes | Yes | INS | No | No | Yes | INS | No | Yes | Yes | No | No | Yes | Yes | Critically low |
|  | DeepSeekR1 | Yes | Yes | Yes | No | Yes | No | No | Yes | No | No | Yes | No | Yes | Yes | Yes | NA | low |
| Hollings et al. 2017 | Human | Yes | PY | Yes | No | Yes | No | No | No | PY | No | Yes | No | No | Yes | No | Yes | Critically low |
|  | ChatGPT4.0 | Yes | No | Yes | Yes | Yes | No | Yes | Yes | No | Yes | No | NA | No | Yes | No | Yes | Critically low |
|  | DeepSeekR1 | Yes | Yes | Yes | Yes | No | No | Yes | Yes | Yes | Yes | No | No | Yes | Yes | No | Yes | Moderate |
| Kraal et al. 2017 | Human | Yes | PY | Yes | No | Yes | No | No | PY | No | No | Yes | No | No | No | Yes | Yes | Critically low |
|  | ChatGPT4.0 | Yes | No | Yes | Yes | PY | Yes | No | Yes | No | PY | Yes | No | No | No | Yes | Yes | Critically low |
|  | DeepSeekR1 | Yes | Yes | Yes | INS | Yes | INS | Yes | No | No | No | Yes | INS | No | Yes | No | Yes | low |
| Lee et al. 2020 | Human | Yes | Yes | No | No | Yes | No | No | PY | Yes | No | Yes | No | No | No | Yes | Yes | Critically low |
|  | ChatGPT4.0 | Yes | No | Yes | Yes | Yes | Yes | No | Yes | No | No | No | Yes | No | No | Yes | No | Critically low |
|  | DeepSeekR1 | Yes | INS | No | No | Yes | Yes | No | Yes | No | INS | Yes | Yes | No | Yes | Yes | Yes | low |
| Liou et al. 2016 | Human | Yes | No | Yes | No | No | No | No | PY | Yes | No | Yes | No | No | No | Yes | No | Critically low |
|  | ChatGPT4.0 | Yes | No | Yes | Yes | No | No | INS | INS | No | No | Yes | Yes | Yes | No | Yes | No | Critically low |
|  | DeepSeekR1 | Yes | INS | Yes | INS | No | INS | Yes | Yes | Yes | INS | Yes | Yes | INS | Yes | No | INS | low |
| Marzolini et al.2012 | Human | Yes | No | Yes | No | Yes | No | No | PY | PY | No | Yes | No | Yes | No | No | Yes | Critically low |
|  | ChatGPT4.0 | Yes | No | Yes | Yes | Yes | No | INS | Yes | INS | No | Yes | No | Yes | No | Yes | Yes | Critically low |
|  | DeepSeekR1 | Yes | No | Yes | No | Yes | No | Yes | Yes | Yes | Yes | No | Yes | Yes | Yes | Yes | No | low |
| Nery et al. 2014 | Human | Yes | No | Yes | PY | Yes | Yes | No | PY | Yes | No | NMA | NMA | No | No | NMA | Yes | Critically low |
|  | ChatGPT4.0 | Yes | No | Yes | Yes | Yes | Yes | No | Yes | Yes | No | No | Yes | No | No | Yes | No | Critically low |
|  | DeepSeekR1 | Yes | No | Yes | Yes | Yes | Yes | No | Yes | Yes | Yes | NA | No | Yes | NA | NA | Yes | low |
| Pattyn et al. 2014 | Human | Yes | No | Yes | No | No | Yes | No | PY | PY | No | Yes | No | Yes | Yes | Yes | Yes | Critically low |
|  | ChatGPT4.0 | Yes | No | Yes | Yes | Yes | Yes | No | Yes | Yes | No | Yes | No | No | No | Yes | Yes | Critically low |
|  | DeepSeekR1 | Yes | INS | Yes | No | Yes | INS | Yes | Yes | No | Yes | Yes | Yes | Yes | Yes | Yes | Yes | Moderate |
| Pattyn et al. 2018 | Human | Yes | No | Yes | No | Yes | Yes | No | PY | PY | No | Yes | No | Yes | Yes | Yes | Yes | Critically low |
|  | ChatGPT4.0 | Yes | No | Yes | Yes | Yes | Yes | No | Yes | Yes | Yes | Yes | Yes | Yes | No | Yes | Yes | Low |
|  | DeepSeekR1 | Yes | No | Yes | No | Yes | Yes | INS | Yes | No | Yes | Yes | No | Yes | No | Yes | INS | Low |
| Qiu et al. 2021 | Human | Yes | No | Yes | No | Yes | Yes | No | PY | Yes | No | Yes | Yes | Yes | Yes | Yes | Yes | Critically low |
|  | ChatGPT4.0 | Yes | No | Yes | Yes | Yes | Yes | No | Yes | Yes | INS | Yes | Yes | Yes | Yes | No | Yes | Low |
|  | DeepSeekR1 | Yes | INS | Yes | No | Yes | No | Yes | Yes | No | Yes | Yes | No | Yes | No | Yes | No | Moderate |
| Schulté et al. 2022 | Human | Yes | PY | Yes | No | Yes | No | No | PY | Yes | No | NMA | NMA | Yes | No | NMA | Yes | Critically low |
|  | ChatGPT4.0 | Yes | Yes | Yes | Yes | Yes | Yes | Yes | Yes | Yes | No | NA | NA | Yes | No | Yes | Yes | Moderate |
|  | DeepSeekR1 | Yes | Yes | No | No | Yes | No | Yes | Yes | No | No | No | Yes | NA | NA | No | No | Critically low |
| Uddin et al. 2016 | Human | Yes | No | Yes | No | Yes | Yes | No | PY | Yes | No | Yes | Yes | Yes | Yes | Yes | Yes | Critically low |
|  | ChatGPT4.0 | Yes | No | Yes | Yes | INS | INS | No | Yes | Yes | No | Yes | No | Yes | No | No | Yes | Critically low |
|  | DeepSeekR1 | Yes | No | Yes | Yes | No | No | No | Yes | Yes | Yes | Yes | Yes | Yes | Yes | Yes | Yes | Critically low |
| Valkeinen et al. 2010 | Human | Yes | No | Yes | Yes | No | Yes | No | PY | PY | No | Yes | No | No | No | Yes | No | Critically low |
|  | ChatGPT4.0 | Yes | No | Yes | Yes | No | Yes | Yes | Yes | Yes | No | Yes | Yes | Yes | No | Yes | Yes | Critically low |
|  | DeepSeekR1 | Yes | No | Yes | No | Yes | No | Yes | PY | No | Yes | Yes | No | Yes | Yes | No | No | low |
| Wang et al. 2022 | Human | Yes | Yes | Yes | PY | Yes | Yes | No | PY | No | No | Yes | No | No | No | Yes | Yes | Critically low |
|  | ChatGPT4.0 | Yes | No | Yes | Yes | Yes | Yes | No | Yes | No | Yes | No | No | No | No | Yes | Yes | Critically low |
|  | DeepSeekR1 | Yes | Yes | No | Yes | Yes | No | Yes | Yes | No | Yes | No | Yes | Yes | No | Yes | Yes | Moderate |
| Xanthos et al. 2017 | Human | No | No | Yes | No | Yes | No | No | PY | Yes | No | No | No | No | No | No | Yes | Critically low |
|  | ChatGPT4.0 | Yes | No | Yes | Yes | Yes | No | Yes | Yes | Yes | No | No | Yes | No | Yes | No | Yes | Critically low |
|  | DeepSeekR1 | Yes | INS | No | Yes | No | No | No | Yes | No | Yes | Yes | No | No | Yes | No | Yes | low |
| Xie et al. 2017 | Human | Yes | No | Yes | No | Yes | Yes | No | PY | Yes | No | Yes | No | Yes | Yes | Yes | No | Critically low |
|  | ChatGPT4.0 | Yes | No | Yes | Yes | Yes | Yes | No | Yes | Yes | No | Yes | No | Yes | No | Yes | Yes | Critically low |
|  | DeepSeekR1 | Yes | INS | Yes | No | Yes | No | Yes | Yes | No | Yes | Yes | Yes | Yes | Yes | No | No | low |
| Yamamoto et al. 2016 | Human | Yes | No | Yes | PY | Yes | No | No | PY | Yes | No | No | No | No | Yes | Yes | No | Critically low |
|  | ChatGPT4.0 | Yes | No | Yes | Yes | Yes | Yes | No | Yes | Yes | No | Yes | Yes | Yes | Yes | Yes | Yes | Critically low |
|  | DeepSeekR1 | Yes | Yes | Yes | No | No | Yes | Yes | Yes | Yes | Yes | Yes | Yes | Yes | Yes | Yes | No | Moderate |
| Zhang et al. 2021 | Human | Yes | No | Yes | PY | Yes | No | No | PY | Yes | No | No | No | No | No | No | Yes | Critically low |
|  | ChatGPT4.0 | Yes | No | Yes | Yes | Yes | Yes | Yes | Yes | Yes | No | Yes | No | Yes | No | Yes | Yes | Critically low |
|  | DeepSeekR1 | Yes | INS | Yes | INS | Yes | INS | Yes | Yes | INS | Yes | No | INS | Yes | Yes | No | INS | Moderate |
| Zheng et al. 2022 | Human | Yes | No | Yes | No | No | Yes | No | PY | Yes | No | Yes | No | No | No | No | Yes | Critically low |
|  | ChatGPT4.0 | Yes | No | Yes | No | INS | No | Yes | Yes | No | Yes | No | Yes | No | NA | No | Yes | Critically Low |
|  | DeepSeekR1 | Yes | Yes | Yes | Yes | Yes | No | Yes | Yes | No | No | Yes | Yes | No | Yes | Yes | Yes | Moderate |

Notes:PY=partial yes;NA=Not Applicable;INS=Insufficient;NMA=no meta-analysis conducted.

1. Did the research questions and inclusion criteria for the review include the components of PICO? 2. Did the report of the review contain an explicit statement that the review methods were established prior to the conduct of the review and did the report justify any significant deviations from the protocol? 3. Did the review authors explain their selection of the study designs for inclusion in the review? 4. Did the review authors use a comprehensive literature search strategy? 5. Did the review authors perform study selection in duplicate? 6. Did the review authors perform data extraction in duplicate? 7. Did the review authors provide a list of excluded studies and justify the exclusions? 8. Did the review authors describe the included studies in adequate detail? 9. Did the review authors use a satisfactory technique for assessing the risk of bias (RoB) in individual studies that were included in the review? 10. Did the review authors report on the sources of funding for the studies included in the review? 11. If meta-analysis was performed did the review authors use appropriate methods for statistical combination of results? 12. If meta-analysis was performed, did the review authors assess the potential impact of RoB in individual studies on the results of the meta-analysis or other evidence synthesis? 13. Did the review authors account for RoB in individual studies when interpreting/ discussing the results of the review? 14. Did the review authors provide a satisfactory explanation for, and discussion of, any heterogeneity observed in the results of the review? 15. If they performed quantitative synthesis did the review authors carry out an adequate investigation of publication bias (small study bias) and discuss its likely impact on the results of the review? 16. Did the review authors report any potential sources of conflict of interest, including any funding they received for conducting the review?

Table 2 Results of large language models and human reviewers in CASP

| References | Tools | 1 | 2 | 3 | 4 | 5 | 6 | 7 | 8 | 9 | 10 | Total |
| --- | --- | --- | --- | --- | --- | --- | --- | --- | --- | --- | --- | --- |
| Nakamura 2002 | Human | 1 | 1 | 1 | 1 | 1 | 1 | 1 | 1 | 1 | 1 | 10 |
|  | ChatGPT4.0 | 1 | 1 | 1 | 0 | 1 | 0 | 1 | 0 | 1 | 1 | 7 |
|  | DeepSeekR1 | 1 | 1 | 1 | 0 | 0 | 0 | 1 | 0 | 1 | 1 | 6 |
| D'alonzo et al. 2008 | Human | 1 | 1 | 1 | 1 | 1 | 1 | 1 | 1 | 1 | 1 | 10 |
|  | ChatGPT4.0 | 1 | 1 | 1 | 0 | 1 | 0 | 0 | 1 | 1 | 1 | 7 |
|  | DeepSeekR1 | 1 | 1 | 1 | 0 | 1 | X | 1 | 0 | 1 | 1 | 7 |
| Sabiston et al. 2009 | Human | 1 | 1 | 1 | 1 | 1 | 0 | 1 | 1 | 1 | 1 | 9 |
|  | ChatGPT4.0 | 1 | 1 | 1 | 1 | 1 | 1 | 1 | 1 | 1 | 1 | 10 |
|  | DeepSeekR1 | 1 | 1 | 1 | 1 | 1 | 1 | 1 | 1 | 1 | 1 | 10 |
| Jiwani et al. 2010 | Human | 1 | 1 | 1 | 1 | 1 | 1 | 0 | 1 | 1 | 1 | 9 |
|  | ChatGPT4.0 | 1 | 1 | 1 | 1 | 1 | 1 | 1 | 1 | 1 | 1 | 10 |
|  | DeepSeekR1 | 1 | 1 | 1 | 1 | 1 | 1 | 1 | 0 | 1 | 1 | 9 |
| O'Dougherty et al. 2010 | Human | 1 | 1 | 1 | 1 | 1 | 0 | 1 | 1 | 1 | 1 | 9 |
|  | ChatGPT4.0 | 1 | 1 | 1 | 1 | 1 | 1 | 1 | 1 | 1 | 1 | 10 |
|  | DeepSeekR1 | 1 | 1 | 1 | 1 | 1 | 0 | 1 | 0 | 1 | 1 | 8 |
| Caperchione et al. 2011 | Human | 1 | 1 | 1 | 1 | 1 | 1 | 1 | 1 | 1 | 1 | 10 |
|  | ChatGPT4.0 | 1 | 1 | 1 | 1 | 1 | 0 | 1 | 1 | 1 | 1 | 9 |
|  | DeepSeekR1 | 1 | 1 | 1 | 1 | 1 | X | 1 | 0 | 1 | 1 | 8 |
| McGannon et al. 2013 | Human | 1 | 1 | 1 | 1 | 1 | 1 | 1 | 1 | 1 | 1 | 10 |
|  | ChatGPT4.0 | 1 | 1 | 1 | 1 | 1 | 1 | 1 | 1 | 1 | 1 | 10 |
|  | DeepSeekR1 | 1 | 1 | 1 | 0 | 1 | X | 1 | 0 | 1 | 1 | 7 |
| Siefken et al. 2014 | Human | 1 | 1 | 1 | 1 | 1 | 0 | 1 | 1 | 1 | 1 | 9 |
|  | ChatGPT4.0 | 1 | 1 | 1 | 1 | 1 | 0 | 1 | 1 | 1 | 1 | 9 |
|  | DeepSeekR1 | 1 | 1 | 1 | 0 | 1 | X | 1 | 0 | 1 | 1 | 7 |
| Walseth et sl. 2014 | Human | 1 | 1 | 1 | 1 | 1 | 1 | 1 | 1 | 1 | 1 | 10 |
|  | ChatGPT4.0 | 1 | 1 | 0 | x | 0 | X | X | 0 | 1 | 1 | 4 |
|  | DeepSeekR1 | 1 | 1 | 1 | 0 | 1 | 0 | X | 0 | 1 | 1 | 6 |
| Alvarado et al. 2015 | Human | 1 | 1 | 1 | 1 | 1 | 0 | 1 | 1 | 1 | 1 | 9 |
|  | ChatGPT4.0 | 1 | 1 | 1 | 1 | 1 | 1 | 1 | 1 | 1 | 1 | 10 |
|  | DeepSeekR1 | 1 | 1 | 1 | 0 | 1 | 0 | 1 | 0 | 1 | 1 | 7 |
| Dave et al. 2015 | Human | 1 | 1 | 1 | 1 | 1 | 1 | 0 | 1 | 1 | 1 | 9 |
|  | ChatGPT4.0 | 1 | 1 | 1 | 1 | 1 | 1 | 1 | 1 | 1 | 1 | 10 |
|  | DeepSeekR1 | 1 | 1 | 1 | 0 | 1 | 0 | X | 1 | 1 | 1 | 7 |
| Miles et al. 2016 | Human | 1 | 1 | 1 | 1 | 1 | 1 | 0 | 1 | 1 | 1 | 9 |
|  | ChatGPT4.0 | 1 | 1 | 1 | 1 | 1 | 1 | 1 | 1 | 1 | 1 | 10 |
|  | DeepSeekR1 | 1 | 1 | 1 | 0 | 1 | 1 | 0 | 0 | 1 | 1 | 7 |
| Sand et al. 2017 | Human | 1 | 1 | 1 | 1 | 1 | 0 | 1 | 1 | 1 | 1 | 9 |
|  | ChatGPT4.0 | 1 | 1 | 1 | 1 | 1 | 0 | 1 | 1 | 1 | 1 | 9 |
|  | DeepSeekR1 | 1 | 1 | 1 | 0 | 1 | X | 1 | 1 | 1 | 1 | 8 |
| Crino et al. 2018 | Human | 1 | 1 | 1 | 1 | 1 | 0 | 1 | 0 | 1 | 1 | 8 |
|  | ChatGPT4.0 | 1 | 1 | 1 | 1 | 1 | 0 | 1 | 1 | 1 | 1 | 9 |
|  | DeepSeekR1 | 1 | 1 | 1 | 1 | 1 | 0 | 1 | 1 | 1 | 1 | 9 |
| Ware et al. 2019 | Human | 1 | 1 | 1 | 1 | 1 | 1 | 1 | 1 | 1 | 1 | 10 |
|  | ChatGPT4.0 | 1 | 1 | 1 | 1 | 1 | 0 | 1 | 1 | 1 | 1 | 9 |
|  | DeepSeekR1 | 1 | 1 | 1 | 1 | 1 | 0 | 1 | 1 | 1 | 1 | 9 |
| Almaqhawi 2021 | Human | 1 | 1 | 1 | 1 | 1 | 1 | 1 | 1 | 1 | 1 | 10 |
|  | ChatGPT4.0 | 1 | 1 | 1 | 1 | 1 | 0 | 1 | 1 | 1 | 1 | 9 |
|  | DeepSeekR1 | 1 | 1 | 1 | 0 | 0 | X | 1 | 1 | 1 | 1 | 7 |
| Bhatnagar et al. 2021 | Human | 1 | 1 | 1 | 1 | 1 | 1 | 0 | 1 | 1 | 1 | 9 |
|  | ChatGPT4.0 | 1 | 1 | 1 | 1 | 1 | 1 | 1 | 1 | 1 | 1 | 10 |
|  | DeepSeekR1 | 1 | 1 | 1 | 1 | 1 | 1 | 1 | 0 | 1 | 1 | 9 |
| Lansburgh et al. 2022 | Human | 1 | 1 | 1 | 1 | 1 | 0 | 1 | 1 | 1 | 1 | 9 |
|  | ChatGPT4.0 | 1 | 1 | 1 | 1 | 1 | 1 | 1 | 1 | 1 | 1 | 10 |
|  | DeepSeekR1 | 1 | 1 | 1 | 1 | 1 | 0 | 1 | 1 | 1 | 1 | 9 |

Notes:1= present in study; 0=unclear in the study; X=absent from the study.

1.Was there a clear statement of the aims of the research?2.Is a qualitative methodology appropriate?3.Was the research design appropriate to address the aims of the research?4.Was the recruitment strategy appropriate to the aims of the research?5.Was the data collected in a way that addressed the research issue?6.Has the relationship between researcher and participants been adequately considered?7.Have ethical issues been taken into consideration?8.Was the data analysis sufficiently rigorous?9. Is there a clear statement of findings?10.How valuable is the research?

Table 3 Results of large language models and human reviewers in PEDro

| References | Tools | 1* | 2 | 3 | 4 | 5 | 6 | 7 | 8 | 9 | 10 | 11 | Total | Quality |
| --- | --- | --- | --- | --- | --- | --- | --- | --- | --- | --- | --- | --- | --- | --- |
| Bishop et al. 2001 | Human | Y | Y | N | Y | N | N | N | Y | Y | Y | Y | 6 | Good |
|  | ChatGPT4.0 | Y | Y | N | Y | N | N | N | Y | N | Y | Y | 5 | Fair |
|  | DeepSeekR1 | Y | Y | N | Y | N | N | N | Y | Y | Y | Y | 6 | Good |
| Bishop et al. 2003 | Human | Y | Y | N | Y | N | N | N | Y | Y | Y | Y | 6 | Good |
|  | ChatGPT4.0 | Y | Y | N | Y | N | N | N | Y | N | Y | Y | 5 | Fair |
|  | DeepSeekR1 | Y | Y | N | Y | N | N | N | Y | Y | Y | Y | 6 | Good |
| Brandenburg 2005 | Human | Y | Y | N | Y | N | N | N | Y | Y | Y | Y | 6 | Good |
|  | ChatGPT4.0 | Y | Y | N | Y | N | N | N | Y | Y | Y | Y | 6 | Good |
|  | DeepSeekR1 | Y | Y | N | Y | N | N | N | Y | N | Y | Y | 5 | Fair |
| Cé et al. 2009 | Human | Y | Y | N | Y | N | N | N | Y | Y | Y | Y | 6 | Good |
|  | ChatGPT4.0 | Y | Y | N | Y | N | N | N | Y | N | Y | Y | 5 | Fair |
|  | DeepSeekR1 | Y | Y | N | Y | N | N | N | Y | Y | Y | Y | 6 | Good |
| Cochrane et al. 2008 | Human | Y | Y | N | Y | N | N | N | Y | Y | Y | Y | 6 | Good |
|  | ChatGPT4.0 | Y | Y | N | Y | N | N | N | Y | N | Y | Y | 5 | Fair |
|  | DeepSeekR1 | Y | Y | N | Y | N | N | N | Y | Y | Y | Y | 6 | Good |
| Demura et al. 2011 | Human | Y | N | N | Y | N | N | N | Y | Y | Y | Y | 5 | Fair |
|  | ChatGPT4.0 | Y | Y | N | Y | N | N | N | Y | N | Y | Y | 5 | Fair |
|  | DeepSeekR1 | Y | N | N | Y | N | N | N | Y | Y | Y | Y | 5 | Fair |
| Evans et al. 2002 | Human | Y | Y | N | Y | N | N | N | Y | Y | Y | Y | 6 | Good |
|  | ChatGPT4.0 | Y | Y | N | Y | N | N | N | Y | N | Y | Y | 5 | Fair |
|  | DeepSeekR1 | Y | Y | N | Y | N | N | N | Y | N | Y | Y | 5 | Fair |
| Fradkin et al. 2015 | Human | Y | Y | N | Y | N | N | N | Y | Y | Y | N | 5 | Fair |
|  | ChatGPT4.0 | Y | Y | N | Y | N | N | N | Y | N | Y | Y | 5 | Fair |
|  | DeepSeekR1 | Y | Y | N | Y | N | N | N | Y | N | Y | Y | 5 | Fair |
| Franco et al. 2008 | Human | Y | Y | N | Y | N | N | N | Y | Y | Y | Y | 6 | Good |
|  | ChatGPT4.0 | Y | Y | N | Y | N | N | N | Y | N | Y | Y | 5 | Fair |
|  | DeepSeekR1 | Y | Y | N | Y | N | N | N | Y | Y | Y | Y | 6 | Good |
| Gelen et al. 2012 | Human | Y | Y | N | Y | N | N | N | Y | Y | Y | Y | 6 | Good |
|  | ChatGPT4.0 | Y | Y | N | Y | N | N | N | Y | N | Y | Y | 5 | Fair |
|  | DeepSeekR1 | Y | Y | N | Y | N | N | N | Y | N | Y | Y | 5 | Fair |
| Haag et al. 2010 | Human | Y | N | N | Y | N | N | N | Y | Y | Y | Y | 5 | Fair |
|  | ChatGPT4.0 | Y | Y | N | Y | N | N | N | Y | N | Y | Y | 5 | Fair |
|  | DeepSeekR1 | Y | Y | N | Y | N | N | N | Y | Y | Y | Y | 6 | Fair |
| Higuchi et al. 2013 | Human | Y | N | N | Y | N | N | N | Y | Y | Y | Y | 5 | Fair |
|  | ChatGPT4.0 | Y | Y | N | Y | N | N | N | Y | N | Y | Y | 5 | Fair |
|  | DeepSeekR1 | Y | N | N | Y | N | N | N | Y | N | Y | Y | 4 | Fair |
| Huang et al. 2011 | Human | Y | Y | N | Y | N | N | Y | Y | Y | Y | Y | 7 | Good |
|  | ChatGPT4.0 | Y | Y | N | Y | N | N | Y | Y | N | Y | Y | 6 | Good |
|  | DeepSeekR1 | Y | Y | N | Y | N | N | Y | Y | N | Y | Y | 6 | Good |
| Ingham et al. 2014 | Human | Y | Y | N | Y | N | N | N | Y | Y | Y | Y | 6 | Good |
|  | ChatGPT4.0 | Y | Y | N | Y | N | N | N | Y | N | Y | Y | 5 | Fair |
|  | DeepSeekR1 | Y | Y | N | Y | N | N | N | Y | Y | Y | Y | 6 | Good |
| Khamwong et al. 2012 | Human | Y | Y | N | Y | N | N | N | Y | Y | Y | Y | 6 | Good |
|  | ChatGPT4.0 | Y | Y | N | Y | N | N | N | Y | N | Y | Y | 5 | Fair |
|  | DeepSeekR1 | Y | Y | N | Y | N | N | N | Y | N | Y | Y | 5 | Fair |
| Knudson et al. 2004 | Human | Y | N | N | V | N | N | N | Y | Y | Y | Y | 4 | Fair |
|  | ChatGPT4.0 | Y | Y | N | Y | N | N | N | Y | N | Y | Y | 5 | Fair |
|  | DeepSeekR1 | Y | N | N | Y | N | N | N | Y | Y | Y | Y | 5 | Fair |
| Molacek et al. 2010 | Human | Y | Y | N | Y | N | N | N | Y | Y | Y | Y | 6 | Good |
|  | ChatGPT4.0 | Y | Y | N | Y | N | N | N | Y | N | Y | Y | 5 | Fair |
|  | DeepSeekR1 | Y | Y | N | Y | N | N | N | Y | Y | Y | Y | 6 | Good |
| Montoya et al. 2009 | Human | Y | Y | N | Y | N | N | N | Y | Y | Y | Y | 6 | Good |
|  | ChatGPT4.0 | Y | Y | N | Y | N | N | N | Y | N | Y | Y | 5 | Fair |
|  | DeepSeekR1 | Y | Y | N | Y | N | N | N | Y | N | Y | Y | 5 | Fair |
| Moran et al. 2008 | Human | Y | N | N | Y | N | N | N | Y | Y | Y | Y | 5 | Fair |
|  | ChatGPT4.0 | Y | Y | N | Y | N | N | N | Y | N | Y | Y | 5 | Fair |
|  | DeepSeekR1 | Y | Y | N | Y | N | N | N | N | Y | Y | Y | 5 | Fair |
| Nepocatych et al. 2010 | Human | Y | N | N | Y | N | N | N | Y | Y | Y | Y | 5 | Fair |
|  | ChatGPT4.0 | Y | Y | N | Y | N | N | N | Y | N | Y | Y | 5 | Fair |
|  | DeepSeekR1 | Y | N | N | Y | N | N | N | Y | N | Y | Y | 4 | Fair |
| Nosaka et al. 2004 | Human | Y | Y | N | Y | N | N | N | Y | Y | Y | Y | 6 | Good |
|  | ChatGPT4.0 | Y | Y | N | Y | N | N | N | Y | N | Y | Y | 5 | Fair |
|  | DeepSeekR1 | Y | Y | N | Y | N | N | N | Y | N | Y | Y | 5 | Fair |
| Otsuji et al. 2002 | Human | Y | Y | N | Y | N | N | N | Y | Y | Y | Y | 6 | Good |
|  | ChatGPT4.0 | Y | N | N | Y | N | N | N | Y | N | Y | Y | 4 | Fair |
|  | DeepSeekR1 | Y | N | N | Y | N | N | N | Y | Y | Y | Y | 5 | Fair |
| Sedgwick and Whalen 2015 | Human | Y | N | N | N | N | N | N | Y | Y | Y | Y | 4 | Fair |
|  | ChatGPT4.0 | Y | N | N | Y | N | N | N | Y | N | Y | Y | 4 | Fair |
|  | DeepSeekR1 | Y | N | N | N | N | N | N | Y | N | Y | Y | 3 | Poor |
| Southard and Groomer 2013 | Human | Y | Y | N | N | N | N | N | Y | Y | Y | N | 4 | Fair |
|  | ChatGPT4.0 | Y | Y | N | Y | N | N | N | Y | N | Y | Y | 5 | Fair |
|  | DeepSeekR1 | Y | Y | N | N | N | N | N | Y | Y | Y | Y | 5 | Fair |
| Symons et al. 2004 | Human | Y | Y | N | Y | Y | N | N | Y | Y | Y | Y | 7 | Good |
|  | ChatGPT4.0 | Y | Y | N | Y | Y | N | N | Y | N | Y | Y | 6 | Good |
|  | DeepSeekR1 | Y | Y | N | Y | N | N | N | Y | Y | Y | Y | 6 | Good |
| Takizawa et al. 2011 | Human | Y | Y | N | Y | N | N | N | Y | Y | Y | Y | 6 | Good |
|  | ChatGPT4.0 | Y | Y | N | Y | N | N | N | Y | N | Y | Y | 5 | Fair |
|  | DeepSeekR1 | Y | Y | N | Y | N | N | N | Y | Y | Y | Y | 6 | Good |
| Torres et al. 2008 | Human | Y | Y | N | Y | N | N | N | Y | Y | Y | Y | 6 | Good |
|  | ChatGPT4.0 | Y | Y | N | Y | N | N | N | Y | N | Y | Y | 5 | Fair |
|  | DeepSeekR1 | Y | Y | N | Y | N | N | N | Y | Y | Y | Y | 6 | Good |
| Wilcox et al. 2006 | Human | Y | Y | N | Y | N | N | N | Y | Y | Y | Y | 6 | Good |
|  | ChatGPT4.0 | Y | Y | N | Y | N | N | N | Y | N | Y | Y | 5 | Fair |
|  | DeepSeekR1 | Y | Y | N | Y | N | N | N | Y | Y | Y | Y | 6 | Good |

Notes:Y=yes;N=No.*Not included in methodological quality scoring

1. The inclusion criteria of the subjects are specified.2. Subjects are randomly assigned to groups (in crossover studies, the treatment order of subjects is randomized).3. The distribution method is hidden.4. In terms of the most important prognostic indicators, the groups are similar at the baseline.5. Blinding all subjects (blinding).6. Blinding all therapists who perform therapy (blinding).7. Blinding all evaluators measuring at least one primary outcome (blinding).8. More than 85% of participants initially assigned to each group had at least one primary outcome measured.9. All participants with measured outcomes must be treated or controlled according to the allocation protocol, and if this is not the case, then at least one primary outcome should be subject to an "intention-to-treat analysis".10. Statistical results for comparison between groups with at least one primary outcome reported.11. Provide point measurements and variation values for at least one primary outcome.

Table 4 Results of large language models and human reviewers in ROB2

| References | Tools | D1 | D2 | D3 | D4 | D5 | Total |
| --- | --- | --- | --- | --- | --- | --- | --- |
| Barene et al.2014 | Human | low | low | low | low | low | low |
|  | ChatGPT4.0 | Some concerns | Some concerns | Some concerns | low | low | Some concerns |
|  | DeepSeekR1 | Some concerns | Some concerns | Some concerns | low | low | Some concerns |
| Barene et al.2013 | Human | low | Some concerns | low | low | low | Some concerns |
|  | ChatGPT4.0 | Some concerns | low | low | low | low | Some concerns |
|  | DeepSeekR1 | Some concerns | Some concerns | High | low | low | High |
| Krustrup et al.2014 | Human | Some concerns | low | low | low | low | Some concerns |
|  | ChatGPT4.0 | Some concerns | low | Some concerns | low | Some concerns | Some concerns |
|  | DeepSeekR1 | Some concerns | low | low | Some concerns | low | Some concerns |
| Krustrup et al.2010 | Human | Some concerns | low | low | low | low | Some concerns |
|  | ChatGPT4.0 | Some concerns | low | Some concerns | low | Some concerns | Some concerns |
|  | DeepSeekR1 | Some concerns | Some concerns | High | Some concerns | Some concerns | High |
| Krustrup et al.2010 | Human | Some concerns | low | low | low | Some concerns | Some concerns |
|  | ChatGPT4.0 | Some concerns | low | Some concerns | low | Some concerns | Some concerns |
|  | DeepSeekR1 | Some concerns | Some concerns | High | Some concerns | Some concerns | High |
| Larsen et al.2017 | Human | low | low | High | low | low | High |
|  | ChatGPT4.0 | low | low | Some concerns | low | low | Some concerns |
|  | DeepSeekR1 | Some concerns | low | Some concerns | low | low | Some concerns |
| Mohr et al.2015 | Human | Some concerns | low | low | low | Some concerns | Some concerns |
|  | ChatGPT4.0 | Some concerns | low | low | low | Some concerns | Some concerns |
|  | DeepSeekR1 | Some concerns | Some concerns | low | low | Some concerns | Some concerns |
| Randers et al.2010 | Human | Some concerns | low | low | low | low | Some concerns |
|  | ChatGPT4.0 | Some concerns | low | Some concerns | low | Some concerns | Some concerns |
|  | DeepSeekR1 | Some concerns | Some concerns | High | Some concerns | Some concerns | High |
| Skoradal et al.2015 | Human | Some concerns | low | low | low | low | Some concerns |
|  | ChatGPT4.0 | Some concerns | low | low | low | Some concerns | Some concerns |
|  | DeepSeekR1 | Some concerns | Some concerns | low | low | low | Some concerns |
| Uth et al.2015 | Human | low | low | low | low | low | low |
|  | ChatGPT4.0 | Some concerns | low | Some concerns | low | low | Some concerns |
|  | DeepSeekR1 | Some concerns | low | Some concerns | low | low | Some concerns |
| Andersen et al.2014 | Human | High | low | low | low | Some concerns | High |
|  | ChatGPT4.0 | High | low | Some concerns | low | low | High |
|  | DeepSeekR1 | Some concerns | low | Some concerns | low | low | Some concerns |
| Helge et al.2014 | Human | Some concerns | low | low | low | low | Some concerns |
|  | ChatGPT4.0 | Some concerns | low | low | low | low | Some concerns |
|  | DeepSeekR1 | Some concerns | Some concerns | Some concerns | low | low | Some concerns |
| Helge et al.2014 | Human | High | low | High | low | low | High |
|  | ChatGPT4.0 | High | low | Some concerns | low | Some concerns | High |
|  | DeepSeekR1 | High | Some concerns | Some concerns | Some concerns | Some concerns | High |
| Uth et al.2018 | Human | low | Some concerns | low | low | low | Some concerns |
|  | ChatGPT4.0 | High | Some concerns | High | low | Some concerns | High |
|  | DeepSeekR1 | low | High | High | Some concerns | low | High |
| de Sousa et al.2014 | Human | Some concerns | low | low | low | low | Some concerns |
|  | ChatGPT4.0 | Some concerns | low | Some concerns | low | Some concerns | Some concerns |
|  | DeepSeekR1 | Some concerns | Some concerns | High | low | Some concerns | High |
| Helge et al. 2010 | Human | Some concerns | low | High | low | low | High |
|  | ChatGPT4.0 | Some concerns | low | High | low | Some concerns | High |
|  | DeepSeekR1 | Some concerns | High | High | low | Some concerns | High |
| Uth et al.2016 | Human | low | low | low | low | low | low |
|  | ChatGPT4.0 | low | low | Some concerns | Some concerns | low | Some concerns |
|  | DeepSeekR1 | Some concerns | Some concerns | High | Some concerns | low | High |

Notes:D1=Randomisation process.D2=Deviations from the intended interventions.D3=Missing outcome data.D4=Measurement of the outcome.D5=Selection of the reported result.

Suppl.2 Processing-time differences between ChatGPT-4 and DeepSeek R1 in literature quality appraisal

Table 5 Processing-time differences between ChatGPT-4 and DeepSeek R1 in AMSTAR 2

| References | ChatGPT4.0(s) | DeepSeekR1(s) | Deviation(s) |
| --- | --- | --- | --- |
| Bourscheid et al. 2021 | 24.00 | 70.90 | 46.90 |
| Chen & Tang 2021 | 20.63 | 72.21 | 51.58 |
| Chen et al. 2016 | 18.03 | 52.00 | 33.97 |
| Cugusi et al. 2020 | 18.74 | 39.20 | 20.46 |
| Du et al. 2021 | 19.15 | 102.63 | 83.48 |
| Elliott et al. 2015 | 13.76 | 52.32 | 38.56 |
| Fan et al. 2021 | 13.75 | 31.28 | 17.53 |
| Garcia et al. 2019 | 15.12 | 73.43 | 58.31 |
| Gomes-Neto et al. 2017 | 14.15 | 38.80 | 24.65 |
| Hannan et al. 2018 | 16.43 | 68.18 | 51.75 |
| Hollings et al. 2017 | 16.01 | 58.03 | 42.02 |
| Kraal et al. 2017 | 12.83 | 111.69 | 98.86 |
| Lee et al. 2020 | 16.27 | 97.99 | 81.72 |
| Liou et al. 2016 | 17.57 | 51.88 | 34.31 |
| Marzolini et al.2012 | 18.07 | 46.90 | 28.83 |
| Nery et al. 2014 | 22.83 | 77.17 | 54.34 |
| Pattyn et al. 2014 | 15.58 | 36.21 | 20.63 |
| Pattyn et al. 2018 | 17.42 | 55.48 | 38.06 |
| Qiu et al. 2021 | 15.73 | 109.21 | 93.48 |
| Schulté et al. 2022 | 15.52 | 40.75 | 25.23 |
| Uddin et al. 2016 | 19.21 | 82.71 | 63.50 |
| Valkeinen et al. 2010 | 14.03 | 71.74 | 57.71 |
| Wang et al. 2022 | 16.19 | 43.69 | 27.50 |
| Xanthos et al. 2017 | 17.11 | 64.41 | 47.30 |
| Xie et al. 2017 | 17.51 | 61.89 | 44.38 |
| Yamamoto et al. 2016 | 14.94 | 71.63 | 56.69 |
| Zhang et al. 2021 | 16.92 | 73.20 | 56.28 |
| Zheng et al. 2022 | 24.00 | 70.90 | 46.90 |

Table 6 Processing-time differences between ChatGPT-4 and DeepSeek R1 in CASP

| References | ChatGPT4.0(s) | DeepSeekR1(s) | Deviation(s) |
| --- | --- | --- | --- |
| Nakamura 2002 | 25.60 | 47.94 | 22.34 |
| D'alonzo et al. 2008 | 24.65 | 69.02 | 44.37 |
| Sabiston et al. 2009 | 24.80 | 66.97 | 42.17 |
| Jiwani et al. 2010 | 20.90 | 57.04 | 36.14 |
| O'Dougherty et al. 2010 | 23.80 | 85.56 | 61.76 |
| Caperchione et al. 2011 | 20.54 | 34.87 | 14.33 |
| McGannon et al. 2013 | 20.90 | 28.97 | 8.07 |
| Siefken et al. 2014 | 28.21 | 66.89 | 38.68 |
| Walseth et sl. 2014 | 20.27 | 42.08 | 21.81 |
| Alvarado et al. 2015 | 19.38 | 35.51 | 16.13 |
| Dave et al. 2015 | 18.08 | 47.55 | 29.47 |
| Miles et al. 2016 | 21.44 | 157.97 | 136.53 |
| Sand et al. 2017 | 21.01 | 55.04 | 34.03 |
| Crino et al. 2018 | 22.19 | 62.02 | 39.83 |
| Ware et al. 2019 | 22.67 | 52.05 | 29.38 |
| Almaqhawi 2021 | 24.96 | 53.98 | 29.02 |
| Bhatnagar et al. 2021 | 21.58 | 49.80 | 28.22 |
| Lansburgh et al. 2022 | 19.77 | 34.67 | 14.9 |

Table 7 Processing-time differences between ChatGPT-4 and DeepSeek R1 in PEDro

| References | ChatGPT4.0(s) | DeepSeekR1(s) | Deviation(s) |
| --- | --- | --- | --- |
| Bishop et al. 2001 | 25.32 | 31.83 | 6.51 |
| Bishop et al. 2003 | 19.57 | 64.26 | 44.69 |
| Brandenburg 2005 | 22.55 | 53.70 | 31.15 |
| Cé et al. 2009 | 16.48 | 35.29 | 18.81 |
| Cochrane et al. 2008 | 15.03 | 39.53 | 24.50 |
| Demura et al. 2011 | 21.07 | 63.52 | 42.45 |
| Evans et al. 2002 | 18.76 | 42.05 | 23.29 |
| Fradkin et al. 2015 | 16.86 | 37.00 | 20.14 |
| Franco et al. 2008 | 13.41 | 43.79 | 30.38 |
| Gelen et al. 2012 | 21.83 | 39.72 | 17.89 |
| Haag et al. 2010 | 19.68 | 36.98 | 17.30 |
| Higuchi et al. 2013 | 15.59 | 52.60 | 37.01 |
| Huang et al. 2011 | 21.50 | 39.06 | 17.56 |
| Ingham et al. 2014 | 21.22 | 41.91 | 20.69 |
| Khamwong et al. 2012 | 17.34 | 57.48 | 40.14 |
| Knudson et al. 2004 | 19.32 | 43.00 | 23.68 |
| Molacek et al. 2010 | 21.09 | 41.00 | 19.91 |
| Montoya et al. 2009 | 18.86 | 42.81 | 23.95 |
| Moran et al. 2008 | 15.72 | 35.42 | 19.70 |
| Nepocatych et al. 2010 | 14.27 | 42.33 | 28.06 |
| Nosaka et al. 2004 | 15.21 | 50.00 | 34.79 |
| Otsuji et al. 2002 | 24.25 | 36.86 | 12.61 |
| Sedgwick and Whalen 2015 | 13.87 | 49.83 | 35.96 |
| Southard and Groomer 2013 | 20.21 | 39.88 | 19.67 |
| Symons et al. 2004 | 14.46 | 56.71 | 42.25 |
| Takizawa et al. 2011 | 18.98 | 44.00 | 25.02 |
| Torres et al. 2008 | 16.29 | 47.69 | 31.40 |
| Wilcox et al. 2006 | 16.74 | 50.39 | 33.65 |

Table 8 Processing-time differences between ChatGPT-4 and DeepSeek R1 in ROB2

| References | ChatGPT4.0(s) | DeepSeekR1(s) | Deviation(s) |
| --- | --- | --- | --- |
| Barene et al. 2014 | 25.63 | 31.75 | 6.12 |
| Barene et al. 2013 | 23.23 | 61.57 | 38.34 |
| Krustrup et al. 2014 | 23.14 | 29.83 | 6.69 |
| Krustrup et al. 2010 | 19.41 | 48.09 | 28.68 |
| Krustrup et al. 2010 | 18.04 | 36.20 | 18.16 |
| Larsen et al. 2017 | 15.45 | 35.16 | 19.71 |
| Mohr et al. 2015 | 17.28 | 33.30 | 16.02 |
| Randers et al. 2010 | 19.32 | 36.61 | 17.29 |
| Skoradal et al. 2015 | 17.07 | 35.68 | 18.61 |
| Uth et al. 2015 | 16.71 | 34.94 | 18.23 |
| Andersen et al. 2014 | 21.47 | 44.16 | 22.69 |
| Helge et al. 2014 | 21.02 | 54.05 | 33.03 |
| Helge et al. 2014 | 20.65 | 41.08 | 20.43 |
| Uth et al. 2018 | 19.36 | 43.66 | 24.30 |
| de Sousa et al. 2014 | 15.16 | 38.57 | 23.41 |
| Helge et al. 2010 | 16.45 | 44.15 | 27.70 |
| Uth et al. 2016 | 15.82 | 46.87 | 31.05 |
